# Repair and regeneration across the lifespan: an ontogenetic perspective

**DOI:** 10.3389/fragi.2026.1888854

**Published:** 2026-08-24

**Authors:** Hugo A. Barrera-Saldaña, Luis E. Fernández-Garza, Fernando Guillen-Silva, Oscar R. Fajardo-Ramirez, Rafael Francisco Iñigo Pavlovich, Fermín Valenzuela Gómez-Gallardo, David E. Rodriguez-Fuentes, Alexia Y. Avila-Villalobos, Silvia A. Barrera-Barrera, Rodrigo A. Somoza

**Affiliations:** 1 Innbiogem SC, Vitaxentrum, Monterrey, México; 2 Facultad de Ciencias Biológicas, Universidad Autónoma de Nuevo León, Ciudad Universitaria, San Nicolás de Los Garza, México; 3 Facultad de Medicina, Universidad Autónoma de Nuevo León, Monterrey, México; 4 Dirección de Investigación Científica, Laboratorios Columbia, Coyoacán, México; 5 Servicio de Neurología, Hospital Universitario “Dr. José Eleuterio González”, Universidad Autónoma de Nuevo León, Monterrey, México; 6 Laboratorio de Fisiología Molecular y Estructural, Facultad de Ciencias Biológicas, Universidad Autónoma de Nuevo León, Ciudad Universitaria, San Nicolás de Los Garza, México; 7 Universidad Tecnológica de México (UNITEC), Campus Monterrey, San Nicolás de Los Garza, Nuevo León, México; 8 Department of Musculoskeletal Diseases, Ti Orthopedica Institute, Tijuana, Mexico; 9 Academia Nacional de Medicina de México, Committee of Liaison and Dissemination of Knowledge, Ciudad de México, México; 10 Department of Biology, Skeletal Research Center, and Center for Modular Manufacturing of Structural Tissues, Case Western Reserve University, Cleveland, OH, United States

**Keywords:** cellular senescence, DNA repair, epigenetic regulation, fibrosis, ontogeny, regenerative potential, stem cell niche, tissue regeneration

## Abstract

The capacity for tissue repair and regeneration undergoes a profound and progressive decline across the human lifespan, representing a fundamental driver of aging and chronic disease. This review establishes a comprehensive ontogenetic framework by mapping the continuous biological transition from the flawless, scarless regenerative plasticity of embryonic development to the irreversible fibrotic scarring and organ failure characteristic of senescence. We synthesize the hierarchical collapse of reparative networks across multiple biological scales. Importantly, this ontogenetic decline should not be interpreted as a purely degenerative trajectory but rather as a dynamic systems-level reprogramming in which evolutionary trade-offs prioritize tumor suppression, immune surveillance, and reproductive fitness over long-term regenerative fidelity. Recognizing this adaptive reallocation of biological resources reframes aging not simply as failure but as a predictable recalibration of repair hierarchies. At the molecular and cellular levels, the accumulation of genomic instability, unresolvable DNA damage, and mitochondrial dysfunction gradually overwhelms intracellular quality-control mechanisms. Concurrently, epigenetic drift and chronic, low-grade systemic inflammation (“inflammaging”) dismantle the stem cell niche, driving adult stem cell exhaustion and shifting wound healing away from functional tissue replacement toward maladaptive fibrosis. Furthermore, we examine divergent, organ-specific repair trajectories. By contrasting the severe regenerative restrictions of the adult central nervous system and myocardium with the persistent, yet exhaustible, resilience of the liver, we elucidate the unique intrinsic and microenvironmental barriers that impede structural and functional recovery. Finally, we evaluate the clinical paradigm shift from passive management of age-related degeneration to active restoration of tissue integrity. By integrating systemic geroscience—which addresses the global hallmarks of aging—with targeted bioengineering and *in vivo* epigenetic modulation, contemporary regenerative medicine seeks to recreate permissive, youthful microenvironments. Ultimately, mastering these ontogenetic principles holds unprecedented potential to reactivate endogenous repair pathways, mitigate multi-organ collapse, and significantly extend human functional healthspan.

## 1 Introduction

Ontogeny describes the biological continuum of an organism from fertilization through senescence (Tomasello and Gonzalez-Cabrera, 2017). Across this trajectory, the maintenance of life depends in part on the organism’s ability to preserve tissue integrity against continuous internal and environmental stressors (Tomasello and Gonzalez-Cabrera, 2017). Consequently, the repair mechanisms are not static; they evolve dynamically to meet the changing physiological demands of each life stage.

From the embryonic stage onward, repair mechanisms are critical for survival (Zhao et al., 2021). Embryos exhibit a unique capacity for highly efficient, often scar-free regeneration, a plasticity that gradually diminishes during fetal and neonatal development. Childhood and adolescence remain periods of robust regenerative potential, supported by active neurogenesis and efficient tissue renewal (Kuipers et al., 2015). However, as the organism transitions into adulthood, this capacity becomes constrained. Although adult stem cells retain some repair potential, their function is increasingly limited by reduced proliferation and altered responses to intrinsic and extrinsic cues.

This decline accelerates with aging. Repair efficiency is compromised by structural changes in the extracellular matrix and by epigenetic drift (Fulop et al., 2018; Heiss et al., 2005), while tissue resilience is further eroded by inflammaging and systemic hormonal and metabolic alterations (Oh et al., 2014; Kaastrup and Grønbæk, 2021; Gethin et al., 2022). Ultimately, in old age, the accumulation of genomic instability, mitochondrial dysfunction, and proteostasis loss overwhelm the body’s repair capacity, driving functional decline across organ systems.

Although existing literature often addresses these mechanisms in isolation—focusing on DNA repair, stem cell biology, or immunosenescence—an integrated perspective is needed to understand how these systems interact over time. This review aims to bridge the gap by exploring the ontogenetic evolution of repair mechanisms. By contrasting the near-flawless regeneration of the embryo with the systemic decline alongside aging, we provide a comprehensive framework relevant to regenerative medicine, developmental biology, and aging research.

To construct this framework, we adopted a narrative review strategy to synthesize the current state of knowledge regarding the drivers of ontogenetic decline and their impact on the regenerative or self-repair capacity of the human body across the lifespan. This design was selected because it permits broader and more comprehensive integration of evidence across multiple biological scales than the more restrictive scope of a systematic review or meta-analysis (Sukhera, 2022). A comprehensive literature search was conducted using PubMed/MEDLINE, Scopus, and the Web of Science. The search strategy utilized combinations of terms related to ontogenetic regenerative decline across distinct biological categories, including genomic and epigenetic factors (epigenetics, DNA repair, DNA methylation, and DNA damage), intracellular mechanisms (proteostasis, organellar repair, redox homeostasis, inducible stress response, cellular senescence, and systemic communication), cell-mediated regeneration (mesenchymal stem cells, tissue repair/regeneration, differentiation potential, secretome, and extracellular vesicles), tissue-specific wound repair (tissue repair, wound healing, tissue regeneration, extracellular matrix remodeling, fibroblast migration, angiogenesis, re-epithelialization, collagen synthesis, growth factors, and matrix metalloproteinases), systemic repair (endocrine regulation, circadian rhythms, glymphatic system, immunosenescence, systemic senescence, and immune surveillance), and geroscience (aging biomarkers, hallmarks of aging, clinical translation, epigenetic clocks, inflammaging, immunosenescence, gut microbiome, mTOR, metformin, senolytics, exercise, sarcopenia, sleep regularity, heart rate variability, sauna bathing, photobiomodulation, and epigenetic reprogramming). To meet the inclusion criteria, we selected peer-reviewed articles evaluating human or mammalian *in vivo* and *in vitro* models. Data from the selected studies were conceptually synthesized to delineate the current understanding of regenerative decline, highlight organ-specific trajectories, and identify gaps in contemporary geroscience.

## 2 Genomic integrity and epigenetic regulation of repair

The integrity of the regenerative response is fundamentally established within the nucleus, where DNA damage repair mechanisms are intrinsically coupled with the epigenetic landscape. Together, these systems determine the accessibility and activation of transcriptional networks required for cellular proliferation and tissue restoration (López-Otín et al., 2023). This equilibrium is further anchored by telomeres, which are specialized nucleoprotein structures that safeguard chromosomal ends. Telomeres prevent terminal DNA from being erroneously recognized as double-strand breaks, a function mediated by the shelter in complex (Chakravarti et al., 2021). As telomeres progressively shorten with each replicative cycle—a process accelerated by oxidative stress—they reach a critical threshold that triggers a persistent DNA damage response (DDR). This signaling not only halts cellular proliferation but also reinforces an epigenetic state conducive to permanent cellular senescence, thereby limiting the long-term regenerative potential of the tissue (Roake and Artandi, 2020).

As organisms age, the fidelity of the genomic repair machinery declines, leading to the accumulation of DNA lesions that drive cellular senescence. Simultaneously, epigenetic drift alters chromatin architecture, thus silencing critical regenerative genes while activating pro-inflammatory cascades (Feil and Fraga, 2012). Therefore, understanding repair at the genomic level requires a unified perspective on both structural DNA maintenance and dynamic epigenetic regulation.

### 2.1 Molecular determinants of DNA damage

DNA damage repair constitutes a fundamental biological safeguard for genomic integrity, preventing the accumulation of mutations that drive degenerative changes, aging, and cancer (Sancar et al., 2004). The mammalian genome operates under a constant siege from both endogenous reactive metabolites and diverse exogenous agents (Christmann et al., 2003). If left unrepaired, the lesions formed compromise the genomic stability and accelerate disease progression (Friedberg et al., 2006).

#### 2.1.1 Endogenous drivers of genomic instability

The primary threat to the genome arises from within. The inherent chemical instability of DNA, coupled with cellular metabolic byproducts, generates a continuous burden of lesions. During replication, despite the high fidelity and proofreading capabilities of DNA polymerases δ and ε, base substitutions and insertions occur at a rate of 10^–6^ to 10^–8^ per cell generation (Kunkel, 2004; 2009). These errors are compounded by the spontaneous hydrolysis of N-glycosylic bonds, which creates between 10,000 and 50,000 apurinic/apyrimidinic (AP) sites per cell each day (Lindahl and Nyberg, 1972; Nakamura and Swenberg, 1999). Furthermore, aerobic metabolism produces reactive oxygen species (ROS) that, while essential for signaling, inflict oxidative stress when dysregulated. ROS-induced lesions, such as 8-oxo-7,8-dihydro-2′-deoxyguanosine (8-oxo-dG), are particularly damaging because they promote mutagenic transversions associated with aging (de Almeida et al., 2022).

#### 2.1.2 Exogenous mutagens and environmental insults

External agents introduce distinct, structurally complex forms of damage. Ultraviolet (UV) radiation represents the most prevalent exogenous mutagen, inducing up to 100,000 lesions per cell daily, primarily in the form of cyclobutane pyrimidine dimers (CPDs) that physically block transcription and replication (Ciccia and Elledge, 2010; Pfeifer, 2020). Similarly, ionizing radiation (IR) and environmental toxins act as potent genotoxins. IR generates both direct strand breaks and indirect oxidative damage via water radiolysis (Ward, 1988), while chemical carcinogens—such as aromatic amines and polycyclic aromatic hydrocarbons (PAHs)—form bulky DNA adducts that distort the helical structure and impede the replicative machinery (Perera et al., 2005; Vineis and Pirastu, 1997).

### 2.2 DNA damage response fidelity and its ontogenetic erosion

Cells with damaged DNA typically activate highly efficient repair mechanisms that restore the original sequence in most cases. Despite the overall fidelity of DNA repair systems, errors can occur, leading to point mutations, insertions, deletions, or large-scale genomic rearrangements that persist for the lifetime of the cell. Moreover, the capacity for DNA damage detection and repair is not constant throughout life. DDR efficiency and repair fidelity follow a predictable trajectory, transitioning from high-fidelity mechanisms in fetal and neonatal stages to more error-prone senescence-associated processes in aged tissues. This progressive decline in genomic maintenance is a key driver of stem cell dysfunction and the reduced regenerative capacity that characterizes biological aging.

#### 2.2.1 Direct reversal, excision, and mismatch repair: age-dependent erosion of base-level genomic fidelity

The simplest form of repair involves the direct enzymatic reversal of lesions, such as the removal of alkyl groups by O^6^-alkylguanine DNA alkyltransferase (AGT) or the oxidative demethylation of bases by AlkB dioxygenases (Ahmad et al., 2015; Kuznetsov et al., 2021). Most DNA damage requires excision, and the efficiency of these excision pathways declines measurably across the lifespan. Small, non-bulky lesions, including those generated by oxidative stress, are processed by base excision repair (BER), and this pathway declines with age (Brooks et al., 2013). BER is an important pathway for maintaining genome integrity in male germ cells, and reduced activity of this pathway is observed with age, contributing to the increased mutant frequency seen in male germ cells from old mice (Intano et al., 2002). In young organisms, DNA polymerase β and APE1 (both involved in BER) are upregulated in response to genotoxic stress, enabling a rapid and proportionate repair response. With aging, this inducibility is lost (Cabelof et al., 2006). The consequences extend beyond mutational accumulation: inefficient BER leads to the persistence of cytotoxic repair intermediates, which have been mechanistically linked to neuronal dysfunction and neurodegeneration in post-mitotic tissues (SenGupta et al., 2021). Bulky, helix-distorting lesions such as UV photoproducts are targeted by nucleotide excision repair (NER), and its capacity also declines with age across multiple tissues, further reducing the organism’s ability to clear mutagenic adducts that accumulate over a lifetime of environmental exposure (Goukassian et al., 2000; Moriwaki et al., 1996). Replicative fidelity is additionally safeguarded by mismatch repair (MMR), which identifies and corrects erroneous insertions or deletions that escape DNA polymerase proofreading, and it is indispensable for microsatellite stability (Liu et al., 2017; Krichevsky et al., 2004). Critically, MMR efficiency declines with aging: studies in human T-cell clones demonstrate a measurable reduction in MMR activity with *in vitro* aging, and microsatellite instability increases progressively with organismal age (Annett et al., 2005). This age-dependent erosion of MMR likely contributes significantly to the mutational accumulation that underlies both malignant transformation and the functional decline of long-lived stem cell populations. Taken together, the coordinated deterioration of direct reversal, BER, NER, and MMR across the lifespan constitutes a progressive failure of base-level genomic surveillance --one that shifts the cellular environment from faithful repair toward error tolerance, and from regenerative competence toward senescence and dysfunction.

#### 2.2.2 Double-strand break resolution and its ontogenetic transition toward error-prone repair

Double-strand breaks (DSBs) are resolved through two pathways whose relative contributions shift fundamentally across the lifespan: error-prone non-homologous end joining (NHEJ), dominant in G1-phase cells, and high-fidelity homologous recombination (HR) restricted to S/G2 phases (Britton et al., 2013). In young, actively cycling tissues, this division of labor is functionally appropriate: HR dominates in proliferating progenitors where template availability is guaranteed, whereas NHEJ efficiently handles DSBs in non-replicating cells where the mutational cost remains manageable. This balance erodes with age in ways that have direct consequences for stem cell integrity and regenerative capacity. Hematopoietic stem and progenitor cells (HSPCs) occupy a predominantly quiescent state, which structurally limits their access to HR and forces a constitutive reliance on error-prone NHEJ for DSB resolution (Mohrin et al., 2010). Although long-lived hematopoietic progenitors can resist ionizing radiation through robust pro-survival signaling and p53-mediated damage responses, short-lived progenitors are more prone to apoptosis under the same conditions, reflecting fundamentally different repair competencies within the stem cell compartment (Seluanov et al., 2001). Over time, the accumulation of NHEJ-mediated repair errors in quiescent HSPCs drives genomic instability, predisposing these cells to functional decline or premalignant transformation. Defects in DNA repair pathways more broadly impair HSC function under both homeostatic conditions and regenerative stress, progressively depleting the progenitor pool on which tissues depend for renewal (Rossi et al., 2007). The molecular basis of this age-dependent shift is increasingly well characterized. With aging, impaired recruitment of Rad51—the central recombinase of the HR pathway—limits the fidelity of template-directed DSB repair, channeling breaks toward NHEJ by default. Simultaneously, the NHEJ machinery itself deteriorates, with reduced levels of key factors, including XRCC4, Ligase IV, and Ligase III, rendering the pathway not only more mutagenic but also less efficient, leaving a fraction of DSBs unresolved or aberrantly joined (Li et al., 2016). The consequence is a compounding genomic crisis: declining HR fidelity and declining NHEJ efficiency act in concert to accelerate mutational accumulation, drive chromosomal rearrangements, and progressively destabilize the genomes of tissue-resident stem cells. This dual deterioration represents one of the most mechanistically direct links between molecular aging and the erosion of regenerative competence at the tissue level. 

#### 2.2.3 Mitochondrial DNA repair vulnerabilities

Although nuclear DNA benefits from a comprehensive repair toolkit, mitochondrial DNA (mtDNA) is far more vulnerable because of oxidative damage generated by both exogenous and endogenous ROS, particularly because of its proximity to the electron transport chain (ETC) (Ames et al., 1993). Moreover, mtDNA relies almost exclusively on BER to manage oxidative lesions (LeDoux et al., 1992). This limited repair capacity, combined with the proximity of mtDNA to the ROS-generating ETC, leads to a higher mutation rate in the mitochondrial genome, which is a key driver of cellular senescence and organismal aging (Nissanka and Moraes, 2018). In this context, mitochondrial BER activity in rat cerebral cortices declines progressively with age, reaching levels that are approximately 80% lower in 30-month-old animals than in 17-day-old embryos. This marked ontogenetic contrast underscores how profoundly the repair capacity changes across the lifespan (D. W. Lee and Yu, 1990). This decline has been attributed to reduced expression of key repair enzymes, including OGG1, the primary glycosylase responsible for 8-oxoG recognition and excision, and DNA polymerase γ, the sole replicative and repair polymerase in the mitochondrial compartment (Chen et al., 2002).

### 2.3 Epigenetic orchestration of genomic maintenance

Epigenetic modifications provide a dynamic regulatory layer over the genome, determining how and when genes are expressed without altering the underlying DNA sequence. Operating at the interface between the genome and the environment, these modifications govern the transcriptional activity, DNA damage signaling, and chromatin architecture. Consequently, they function as central orchestrators of genomic stability and tissue repair (Fernandez et al., 2021).

Throughout ontogeny, the epigenome dictates not only the spatial localization of repair responses but also their intensity, a plasticity that is highly efficient during development but progressively dysregulated during aging (Jones et al., 2015).

#### 2.3.1 Chromatin remodeling and histone modifications

The physical accessibility of DNA lesions is a fundamental prerequisite for the repair pathways discussed previously. Histone acetylation serves as a critical mechanism by relaxing chromatin and facilitating the recruitment of DNA repair machinery. Histone acetyltransferase (HAT) complexes and ATM-mediated phosphorylation of KAP-1 increase chromatin accessibility at damage sites, while phosphorylation of H2AX (γH2AX) serves as the founding epigenetic signal for damage recognition and repair factor recruitment (Goodarzi et al., 2008; Rogakou et al., 1998). Critically, γH2AX foci accumulate in aged organisms, reflecting the persistence of unrepaired lesions. This distinction implicates declining repair efficiency, not merely elevated genotoxic burden, as a driver of age-associated genomic instability (Sedelnikova et al., 2004). Another chromatin-remodeling mechanism linking aging and DNA repair is mediated by SIRT1, a NAD^+^-dependent deacetylase essential for maintaining repressive chromatin marks at repetitive elements and developmental gene loci. SIRT1 relocalizes to DNA damage sites in response to chronic genotoxic stress, producing transcriptional changes similar to those observed in aged mouse brains (Oberdoerffer et al., 2008). DNA damage sites recruit SIRT1 away from its homeostatic chromatin functions, leading to epigenetic dysregulation, impaired DNA repair, and the progressive accumulation of damage at an accelerated rate. Therefore, declining repair efficiency can be mechanistically rooted in the progressive remodeling of the histone modification landscape with age. In addition to the acetylation changes, aging is accompanied by alterations in histone methylation patterns. Trimethylation marks on histone H3 lysines—H3K4me3, H3K9me3, H3K27me3, and H3K36me3—undergo significant remodeling, often reflecting a decline in heterochromatin organization with age (Pal and Tyler, 2016). Collectively, these age-dependent alterations in histone modification patterns affect the chromatin architecture, which the repair machinery depends on, thus directly compromising genomic maintenance.

#### 2.3.2 DNA methylation, demethylation, and epigenetic memory

Perhaps the most profound epigenetic influence on tissue regeneration lies in the dynamic regulation of DNA methylation. The strong relationship between DNA methylation and aging has been leveraged to develop predictive models of biological age, known as epigenetic clocks. Unlike permanent genetic mutations, methylation at CpG dinucleotides is reversible, allowing cells to adapt their transcriptional programs in response to injury (Singh et al., 2025). DNA methylation is closely associated with DNA damage repair: aberrant methylation patterns promote genomic instability, including mitotic catastrophe in cell lines; mice lacking *Dnmt1* exhibit genetic instability; DNMT1 is recruited to sites of DNA damage; and interactions between DNMTs and PCNA support both DNA replication and repair (Sriraman et al., 2020). Age-associated changes in DNA methylation are characterized by a global decline in methylation levels, which are partly attributed to the progressive reduction of the maintenance of DNA methyltransferase DNMT1. This global hypomethylation is accompanied by locus-specific demethylation at gene promoters. Concurrently, aging is associated with site-specific DNA hypermethylation at particular CpG regions, likely contributing to the repression of selected gene sets (Liu et al., 2011). TP73, a gene that shares functional features with TP53, including the mediation of DNA damage-induced apoptosis, has been reported to be methylated in samples from postmenopausal women aged 52–78 years (Teschendorff et al., 2010). In addition, comparative methylome analyses, such as those performed in CD4^+^ T-cells from newborns and centenarians, further support a model in which aging involves both widespread hypomethylation and increased heterogeneity in DNA methylation patterns across the genome (Heyn et al., 2012). Overall, aging is marked by complex epigenomic remodeling involving both global and locus-specific DNA methylation changes that affect gene expression and cellular function.

#### 2.3.3 Post-transcriptional regulation via non-coding RNAs

Beyond the chromatin structure, repair networks are finely tuned post-transcriptionally by non-coding RNAs (ncRNAs). The diverse regulatory roles of ncRNAs make it difficult to establish consistent expression patterns across aging contexts (Majidinia et al., 2020); yet, specific miRNAs have been directly linked to age-dependent changes in DDR regulation. At the DDR level, miR-29 increases during aging and reinforces p53-dependent DNA damage responses, contributing to persistent damage signaling and the progression of cellular senescence (Turko et al., 2025). Similarly, elevated miR-34 levels observed in aging models target BCL2 and SIRT1, with the inverse correlation between miR-34 and SIRT1 expressions indicating reduced cellular resilience to genotoxic stress during aging (Wang et al., 2009). Given the established role of SIRT1 in chromatin remodeling at damage sites, as described in Section 2.3.1, miR-34-mediated suppression of SIRT1 represents a post-transcriptional mechanism that compounds the epigenetic erosion of DDR capacity with age. Evidence has been accumulating, indicating that age-dependent changes in ncRNA expression constitute an additional regulatory layer through which genomic maintenance capacity is progressively undermined, converging with the chromatin and methylation changes described above to amplify the ontogenetic decline in tissue-repair competence.

### 2.4 Integration of genetic and epigenetic repair networks

**FIGURE 1:**
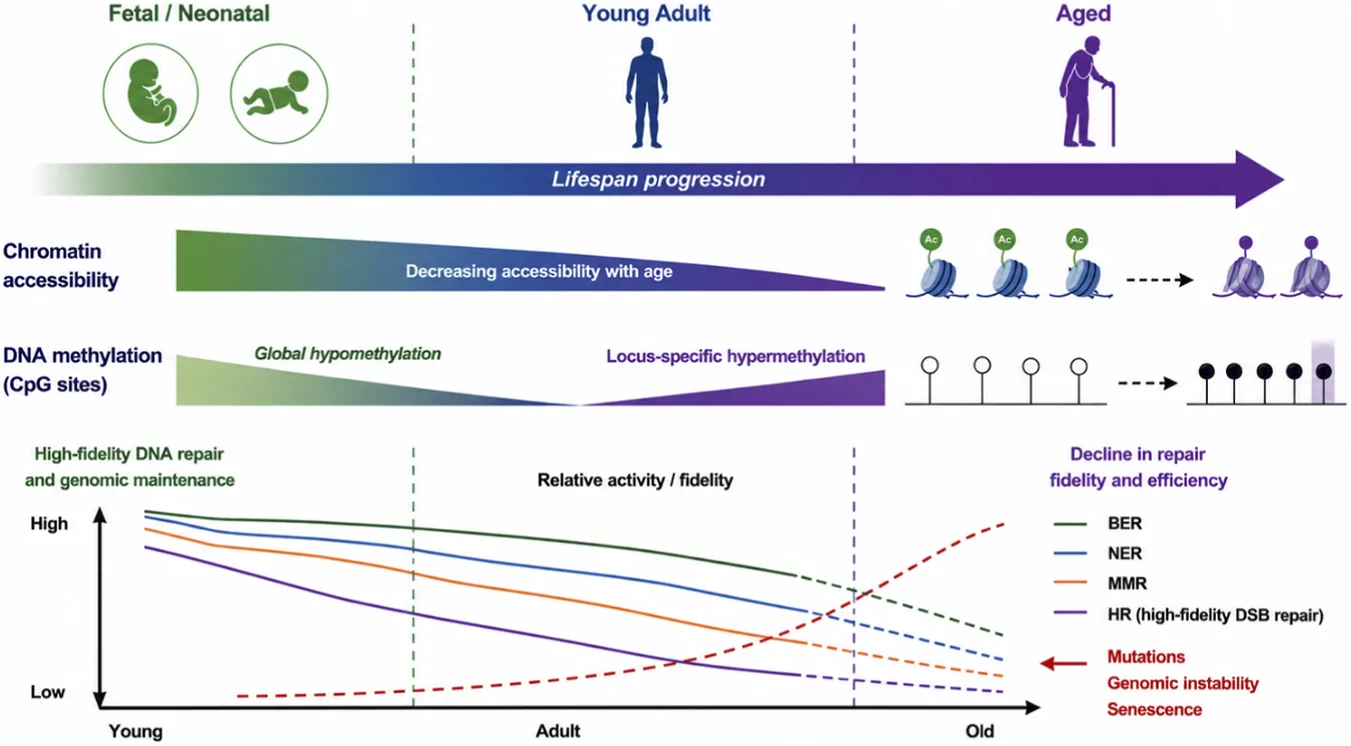
Ontogenetic erosion of DNA damage response. Across the lifespan, the mutational burden increases because of internal and external factors, while DNA repair responses decline markedly. Together, these changes contribute to genomic instability, one of the hallmarks of aging. Epigenetic processes, including chromatin remodeling and global DNA methylation, also undergo progressive modification throughout the lifespan.

Ultimately, DNA methylation, histone modifications, and miRNA regulation do not act in isolation. They operate as an integrated regulatory matrix that allows cells to remember past injuries, modulate genomic accessibility for repair enzymes, and execute complex regenerative programs (Figure 1). However, the fidelity of this epigenetic matrix depends strongly on the developmental stage. As ontogeny progresses toward senescence, the loss of precise epigenetic coordination—manifested as epigenetic drift and depletion of stem cell memory—becomes a primary driver of age-related decline in repair capacity (Fulop et al., 2018; Jones et al., 2015; López-Otín et al., 2013).

## 3 Intracellular homeostasis and quality control mechanisms

Having established how the genome is protected and epigenetically regulated, the next critical scale of the regenerative hierarchy lies within the cytoplasm. Unlike organism-level regeneration, which replaces lost tissues, intracellular repair operates at the molecular and organellar scales. Throughout ontogeny, the efficiency of these systems dictates the cellular lifespan and tissue resilience, transitioning from highly robust networks in embryonic stem cells to progressively compromised systems during aging (Kuzu et al., 2025; Ulfig and Jakob, 2024; Pytel and Fromm Longo, 2025).

### 3.1 The architecture of intracellular quality control and organellar maintenance

The proteostasis network constitutes the foundational tier of this defense architecture. It continuously monitors protein folding, refolds misfolded proteins through molecular chaperones, and targets irreparably damaged proteins for degradation through the ubiquitin–proteasome system (UPS) or autophagy–lysosome pathways.

This network integrates seamlessly with specialized organellar repair. For instance, mitochondria utilize dedicated chaperones, proteases, and dynamic remodeling processes—including fusion, fission, and selective mitophagy—to manage the unique challenges of their dual-genome functionality and highly oxidative environment. In parallel, redox homeostasis mechanisms, principally the glutathione and thioredoxin systems, act as dynamic buffers by reversing oxidative modifications in proteins and neutralizing oxidative damage in lipids.

Finally, physical breaches in cellular compartmentalization are addressed by membrane repair pathways, which use calcium-dependent annexin recruitment and endosomal sorting complex required for transport (ESCRT)-mediated vesicle shedding to restore the lipid bilayer integrity (Elmi and Nylandsted, 2025; Sønder et al., 2019).

### 3.2 Hierarchical defense: from basal maintenance to terminal response

To survive dynamic environmental challenges without continuously expending maximal energy, cells have evolved a tiered defense hierarchy (Masser et al., 2020). At the primary level, basal quality control operates constitutively. These housekeeping functions—such as steady-state autophagy, basal proteasomal degradation, and constitutive antioxidant scavenging—quietly resolve minor everyday stresses without disrupting normal cellular physiology (Korolchuk et al., 2010; Gatica et al., 2018; Dikic and Elazar, 2018).

When insults exceed this basal capacity, cells transition to the second tier: inducible stress responses. These emergency programs are governed by specialized sensor pathways that activate stress-responsive transcription factors. Classic examples include heat shock factor 1 (HSF1) during proteotoxic heat shock, nuclear factor erythroid 2-related factor 2 (NRF2) during oxidative stress, and the unfolded protein response (UPR) in the endoplasmic reticulum (ER) (Ciccarelli and Andréasson, 2024; Pincus, 2020; Zheng et al., 2016). Crucially, these inducible pathways are mechanistically linked to basal systems through feedback loops. In unstressed cells, excess chaperones, such as heat shock proteins 70 and 90 (HSP70/90), sequester HSF1 in a repressed state. As misfolded proteins accumulate, chaperones are redirected to damaged substrates, releasing HSF1 to activate a large compensatory transcriptional program (Anckar and Sistonen, 2011).

If adaptive mechanisms fail to restore homeostasis, the cell engages its ultimate defense tier: terminal responses. The UPR exemplifies this adaptive-to-terminal continuum; while it initially increases ER folding capacity and halts global translation, unresolvable ER stress shifts UPR signaling toward apoptotic pathways (Ghemrawi and Khair, 2020; Hetz et al., 2020). By eliminating the irreversibly damaged unit, the cell effectively sacrifices itself to prevent the propagation of proteotoxic or genotoxic damage to surrounding tissues (Walter and Ron, 2011).

### 3.3 Evolutionary trade-offs and antagonistic pleiotropy

Cellular defenses are evolutionary products optimized for reproductive success and not necessarily for indefinite healthspan. Consequently, mounting these defenses often involves biological trade-offs. The most prominent example is the inverse relationship between tumor suppression and tissue aging. In response to severe DNA damage, the p53 pathway induces either apoptosis or cellular senescence, an irreversible growth arrest (Levine, 1997). Although this mechanism effectively prevents the proliferation of malignant clones in early life, the age-related accumulation of senescent cells becomes deleterious and may increase oncogenic risk.

These cells secrete pro-inflammatory factors—the senescence-associated secretory phenotype (SASP)—which disrupt the tissue microenvironment and actively drive organismal aging (Campisi and d’Adda di Fagagna, 2007; Collado et al., 2007). This phenomenon illustrates antagonistic pleiotropy: a defense mechanism that is highly beneficial during early ontogeny but distinctly harmful in later life.

Similarly, the magnitude of an inducible response must be rigorously regulated. Although acute inflammation or NRF2-mediated antioxidant responses are protective, chronic hyperactivation can cause collateral tissue damage or be hijacked by pathological processes. For example, loss-of-function mutations in Kelch-like ECH-associated protein 1 (*KEAP1*) in cancer cells chronically activate NRF2, granting tumors abnormal resistance to oxidative stress and chemotherapeutics while simultaneously helping them evade immune surveillance (Cyran and Zhitkovich, 2022). Furthermore, overactive innate immune responses can trigger fatal cytokine storms, demonstrating that uncalibrated defense can be more destructive than the initial insult (Tisoncik et al., 2012; Mehta et al., 2020; Paludan et al., 2021).

### 3.4 Cross-scale integration of intracellular defense networks

Intracellular repair does not occur in a vacuum; it is integrated across multiple scales of biological organization. Evidence increasingly demonstrates that proteostasis is coordinated at the organismal level. Transcellular chaperone signaling allows cells experiencing stress, such as specific neurons, to release systemic signals that preemptively upregulate protective networks in distant, unstressed tissues, thereby fortifying the entire organism against impending challenges (Ferreira et al., 2022; Taylor and Dillin, 2011).

This cell-to-system communication is also mediated by immune and neuroendocrine axes. Damaged or necrotic cells release damage-associated molecular patterns (DAMPs), such as high mobility group box 1 (HMGB1) and ATP, which are recognized by immune pattern-recognition receptors (PRRs) to initiate systemic inflammatory and repair cascades (Kono and Rock, 2008; Matzinger, 2002).

Simultaneously, systemic stressors activate the hypothalamic–pituitary–adrenal (HPA) axis, releasing glucocorticoids that tune cellular stress tolerance and immune reactivity across the body (Charmandari et al., 2005). Ultimately, the ontogenetic decline in repair capacity is not merely a failure of isolated intracellular pathways but a breakdown in this sophisticated cross-scale communication, leaving older organisms unable to synchronize a cohesive systemic defense.

### 3.5 Cellular senescence

Cellular senescence is characterized by irreversible cell-cycle arrest accompanied by profound macromolecular changes triggered by diverse stressors. These apoptosis-resistant cells remain viable and metabolically active (Coppé et al., 2010; Kuilman and Peeper, 2009; Muñoz-Espín and Serrano, 2014). Their elevated metabolic state gives rise to the SASP, which is characterized by the release of a wide range of proinflammatory cytokines, chemokines, and growth factors. Physiologically, the SASP can play a beneficial role by promoting immune cell recruitment during development and tissue repair (Demaria et al., 2014; Storer et al., 2013).

However, the persistent accumulation of senescent cells exerts deleterious effects largely through sustained SASP activity, leading to chronic inflammation, tissue dysfunction, fibrosis, and stem cell exhaustion. Abnormal accumulation of senescent cells has been observed not only in aged human tissues but also in tissues from non-human primates and rodents. In early life, the immune system maintains the normal turnover of senescent cells; during aging, this clearance process becomes progressively disrupted.

### 3.6 What determines the lifespan of a cell?

Whether a cell lives for minutes, days, or decades depends on how much damage it accumulates, how effectively it can repair that damage, and which elimination programs are available if repair fails.

One of the strongest determinants of cellular lifespan is the cell identity and differentiation state. Rapidly renewing tissues such as blood, intestinal epithelium, and skin rely on continuous cell replacement. Cells in these tissues often live for only hours to days, not because they are fragile, but because turnover is an efficient strategy for maintaining tissue integrity while limiting the accumulation of mutations (Ajoolabady et al., 2025; Banjac et al., 2023; Flier and Clevers, 2009). In contrast, post-mitotic cells such as neurons and cardiomyocytes are designed for long-term survival. Because replacement is limited or absent, these cells invest heavily in DNA repair pathways, proteostasis, autophagy, and mitochondrial quality control to preserve function over decades (Aranda-Anzaldo, 2012; de Boer et al., 2023; A. Kumar and Thirumurugan, 2023).

The marked differences in lifespan among cell types reflect evolutionary trade-offs. Short-lived immune cells, such as neutrophils, are optimized for rapid response and self-elimination to prevent collateral damage, whereas long-lived neurons prioritize survival but tolerate gradual functional decline (Maklakov and Chapman, 2019). In this sense, cellular lifespan is tuned to tissue-level needs rather than to maximal longevity *per se*.

Importantly, a cell’s fate is not fixed. The nature, intensity, and duration of stress can redirect cellular outcomes. Mild or transient stress can enhance repair and survival, a phenomenon often referred to as hormesis (Zimmermann et al., 2014). In contrast, chronic low-level damage favors entry into cellular senescence. The dynamics of p53 signaling are critical: pulsatile p53 activation tends to promote repair and survival, whereas sustained p53 activation drives senescence or apoptosis (Purvis et al., 2012).

## 4 Cellular plasticity and stem-cell-mediated regeneration

When intracellular quality-control mechanisms, such as proteostasis and mitophagy, are overwhelmed, the biological strategy transitions from internal maintenance to complete cell replacement. This level of defense relies on the organism’s stem cell reservoirs to generate new, functional cellular units that preserve tissue architecture.

This transition from molecular quality control to cellular regeneration represents a fundamental change in repair strategy: rather than repairing damaged components within existing cells, cellular repair generates new, functional cells to replace those lost to injury, disease, or aging (Huyghe et al., 2024; Sharifi et al., 2025).

Cellular repair relies on graded forms of plasticity, ranging from stem cell activation to context-dependent fate flexibility in differentiated cells. This plasticity manifests across an ontogenetic spectrum: from totipotent zygotes capable of generating an entire organism, to multi-potent adult stem cells (ASCs) that replenish specific tissue lineages, to terminally differentiated cells that can, under certain conditions, dedifferentiate and reacquire regenerative potential (Malik and Wang, 2022; Sharifi et al., 2025).

Recent discoveries reveal that cellular plasticity is dynamically regulated by epigenetic modifications. Injury triggers marked changes in chromatin accessibility and DNA methylation that activate regenerative programs in otherwise quiescent cells, enabling them to adapt their differentiation trajectories to meet repair demands (Falick et al., 2022; Yang et al., 2025).

Beyond stem-cell-mediated regeneration, cellular reprogramming has emerged as a powerful experimental and therapeutic paradigm for tissue repair. Transdifferentiation, a form of direct reprogramming, converts one differentiated cell type into another without passing through a pluripotent intermediate.

### 4.1 The ontogenetic gradient of cellular potency and plasticity

Stem cell potency operates along a developmental gradient. During human pre-implantation development, the totipotent zygote undergoes lineage segregation into the trophectoderm, which forms the placenta, and the inner cell mass (ICM), which is the source of pluripotent embryonic stem cells (ESCs) (Condic, 2014; De Paepe et al., 2014; Li et al., 2025).

**FIGURE 2:**
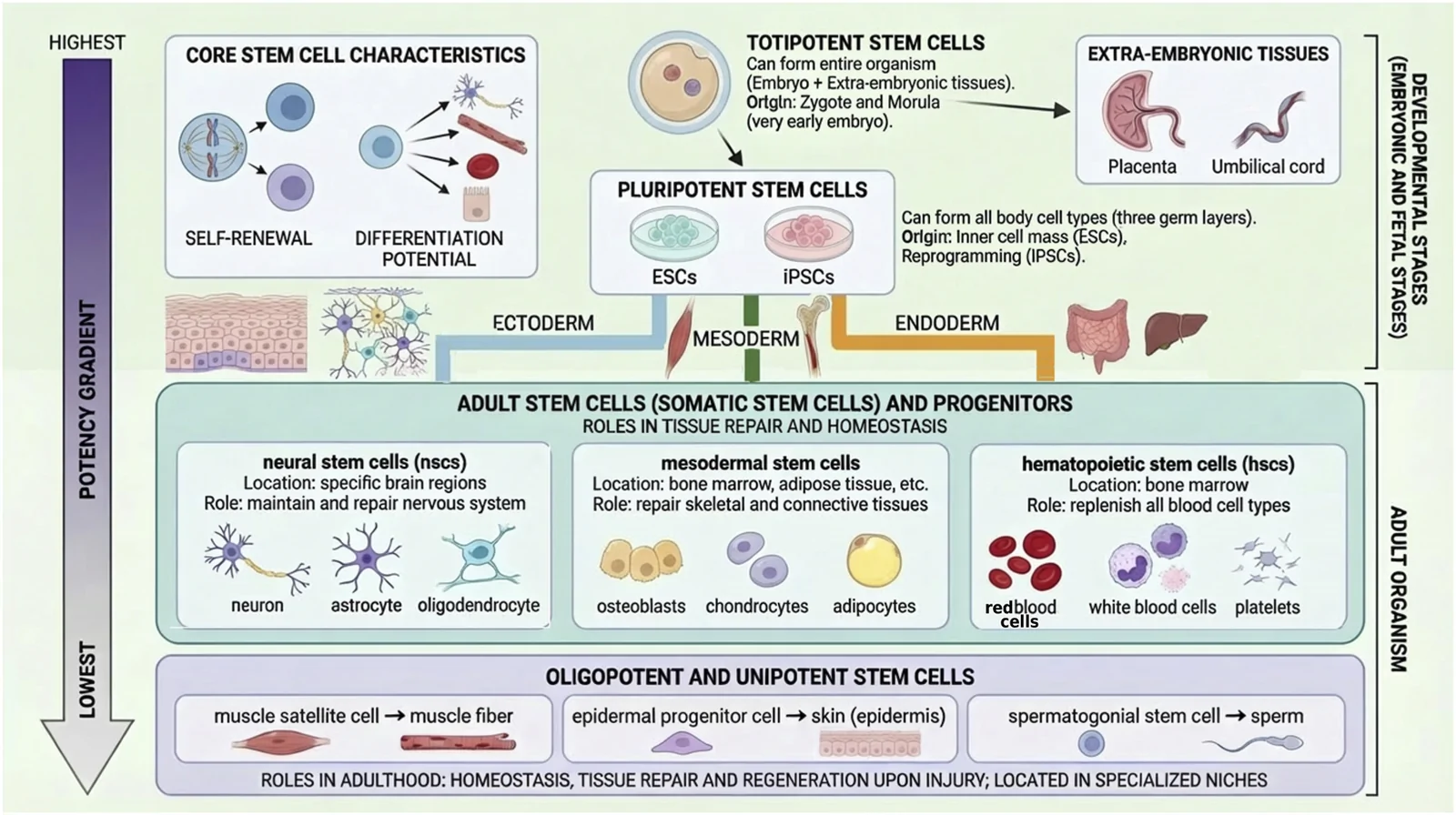
Stem cell hierarchy. General visualization of stem cell potency, classification, and roles from embryonic development to adulthood.

As illustrated in Figure 2, ESCs can differentiate into all three germ layers: the endoderm, which gives rise to the gastrointestinal and respiratory tracts; the ectoderm, which generates the central nervous system and epidermis; and the mesoderm, which forms the musculoskeletal and cardiovascular systems and blood components (Benitah and Frye, 2012; Cheng et al., 2013; Marion and Mao, 2006; Sobhani et al., 2017). As embryonic development concludes, this broad pluripotency becomes progressively restricted. The resulting multipotent ASCs, or somatic stem cells, reside in specific tissue niches where they maintain homeostasis throughout the organism’s lifespan.

A more comprehensive overview of the cellular hierarchy, key phenotypic markers, anatomical niches, differentiation potency, and primary regenerative roles of major stem and progenitor cell populations is provided in Table 1.

**TABLE 1:** Comprehensive stem cell classification.

Classification category	Stem cell type	Key markers	Location/niche	Differentiation potency	Self-renewal capacity	Quiescence	Primary roles	Example lineages/end-stage cells
Types of SCs by potency	Totipotent	Functional state (zygote)	Fertilized egg	All embryonic + extra-embryonic tissues	Indefinite	Actively dividing	Origin of the organism	Zygote → all tissues
	Pluripotent	*OCT4, SOX2,* and *NANOG*	Inner cell mass of blastocyst; iPSCs *in vitro*	All embryonic lineages	Indefinite	Sometimes quiescent	Development, regeneration, and disease modeling	Embryonic stem cells, iPSCs
	Multipotent	Tissue-specific panels (see below)	Organ/tissue niches, often perivascular	Multiple fates (within lineage)	High but limited; may enter into rest	Often quiescent	Lifelong tissue maintenance, a reservoir for regeneration	Adult stem cells (HSCs, NSCs, ISCs, and MSCs)
Not SCs but differentiable	Progenitor cells	Lineage-primed signatures	Intermediate descendants of ASCs	Limited: multipotent → oligopotent → unipotent	Limited divisions	Typically, actively dividing	Rapid proliferation, differentiation for homeostasis, and repair	Myeloid progenitors → granulocytes, macrophages, dendritic cells, and mast cells
Types of adult SCs	HSCs	CD34^+^, CD90^+^, CD49f+, Lin−, CD38^−^, and CD45RA−	Bone marrow	Multipotent	High and lifelong	Often quiescent	Continuous blood production	Myeloid and lymphoid lineages
	NSCs	s100B, nestin, Prox1, GFAP, SOX2, ASCL1, REST, Tbr2, and FOXO3	SVZ, SGZ	Multipotent	High but limited	Often quiescent	Neurogenesis, gliogenesis, and repair	Neurons, astrocytes, and oligodendrocytes
	EPCs	CD31, VEGFR2, CD34, CD146, PDGFRα, CD157, CD200, ABCG2, EPCR, and SCA1	Circulating blood, vessel walls, and placenta	Multipotent (vascular lineages)	Moderate	Variable	Angiogenesis and vascular repair	Endothelial cells
	MuSCs	Pax7, integrin α7, VCAM1, CD34, M-Cadherin, CD82, and CD318	Basal lamina/sarcolemma of muscle fibers	Multipotent (muscle lineages)	High but limited	Quiescent until injury	Skeletal muscle regeneration	Myofibers, smooth muscle cells, and endothelial cells
	ISCs	LGR5 and BMI1	Base of intestinal crypts	Multipotent (epithelial lineages)	High	Actively cycling	Daily epithelial renewal and rapid repair	Enterocytes, goblet cells, and Paneth cells
	Hair follicle stem cells	CD34, K15, SOX9, and LHX2	Bulge region of the hair follicle	Multipotent (epidermal/hair lineages)	High	Quiescent, activated in cycles	Hair cycle and epidermal repair	Keratinocytes and fibroblasts
	Smooth muscle stem cells	SMα-actin (*ACTA2*) and SM22α	Tunica media/adventitia of vessels, airways, and GI tract	Multipotent (smooth muscle lineages)	Moderate	Variable	Vascular tone, remodeling, and repair	Smooth muscle cells
	Spermatogonial stem cells	GFRα1 and PLZF	Basement membrane of seminiferous tubules	Unipotent (sperm lineage)	Lifelong	Quiescent reservoir	Spermatogenesis	Spermatozoa
As SCs are only *in vitro*	MSCs	CD73^+^, CD90^+^, CD105+; CD45^−^, CD34^−^, CD14^−^, CD79^−^, and HLA-DR^−^	Bone marrow, adipose, cord blood, and perivascular niches	Multipotent (mesenchymal lineages)	High but limited	Often quiescent	Stromal support, immune-modulation, and tri-lineage differentiation	Osteoblasts, chondrocytes, and adipocytes
Other adult SCs	VSELs	SSEA, OCT4A, Rex1, and Sca1	Bone marrow, cord blood, and peripheral blood	Claimed pluripotency; definitions debated	Variable	​	Controversial populations. Require functional validation	Reported multilineage differentiation
	MAPCs	CD44, CD73, CD90, CD105, OCT4, and NANOG	Bone marrow	Claimed pluripotency; definitions debated		​	Controversial populations. Require functional validation	
	MUSE		Bone marrow	Claimed pluripotency; definitions debated		​	Controversial populations. Require functional validation	
	MIAMI		Bone marrow	Claimed pluripotency; definitions debated		​	Controversial populations. Require functional validation	

ASCs: adult stem cells; EPCs: endothelial progenitor cells; HSCs: hematopoietic stem cells; ISCs: intestinal stem cells; MAPCs: multipotent adult progenitor cells; MIAMI: marrow-isolated adult multilineage inducible cells; MSCs: mesenchymal stem/stromal cells; MuSCs: muscle satellite cells; MUSE: multilineage-differentiating stress-enduring cells; NSCs: neural stem cells; SCs: stem cells; SGZ: subgranular zone; SVZ: subventricular zone; VSELs: very small embryonic-like stem cells.

Evidence indicates that stem cells modify their behavior, differentiation potential, and reparative functions according to the organism’s developmental stage (see Table 2 in Section 9).

**TABLE 2:** Evolution of regenerative potential across the lifespan.

Biological setting	Fetal	Childhood	Adulthood	Old age
Genomic integrity and epigenetic regulation	High-fidelity DNA repair and genomic maintenance. High global methylation and chromatin accessibility	Not biological settings-specific evidence available currently		Decline in fidelity and efficiency. High burden of mutations. Decrease in global methylation with hypermethylation in specific loci. Less chromatin accessibility
Intracellular homeostasis and quality control mechanisms	High biosynthetic and remodeling capacity; strong chaperone activity, ER quality control, and protein turnover support rapid tissue formation. Developmental metabolism is plastic; redox signaling guides differentiation, while antioxidant systems protect proliferating cells. The intracellular environment favors growth, patterning, and structural assembly; regenerative programs are broadly permissive	Robust but increasingly specialized proteostasis; growth demands are balanced by efficient repair and organelle renewal. Metabolic flexibility remains high; nutrient sensing, mitochondrial turnover, and adaptive stress responses preserve cellular homeostasis. Repair capacity is still strong but progressively constrained by tissue maturation and lineage-specific differentiation	Maintenance predominates overgrowth; proteasome, autophagy, and mitochondrial quality control remain active but become stress-dependent. Repeated metabolic, mechanical, and inflammatory stress increases dependence on AMPK, mTOR, UPR, and integrated stress-response pathways. Regeneration becomes conditional: repair is effective when damage is limited and cellular quality-control systems are preserved	Progressive decline in proteostasis, autophagic flux, mitophagy, and lysosomal efficiency; damaged proteins and organelles accumulate. Mitochondrial dysfunction, oxidative stress, ER stress, and low-grade inflammation impair adaptive responses and tissue resilience. Reduced cellular clearance and chronic stress signaling limit regeneration, promote senescence, and favor fibrosis or incomplete repair
Cellular plasticity and stem-cell-mediated regeneration	Maximum plasticity. Proliferation activity dominates. Chromatin is broadly accessible, allowing rapid lineage commitment. The primary function is to build, not to repair or maintain	The transition from building to maintaining starts to activate. Progressive entry into quiescence. The stem cell niche matures and specializes. Chromatin becomes less accessible, but regeneration-associated enhancers remain active. The primary function is to maintain tissue stability	Quiescent states dominate. Injury response is fully active. Chromatin remodeling is activated upon injury. Lineage memory is retained (plasticity is restricted and is context-dependent). The primary function is to respond to injury and activate repair mechanisms	Quiescence programs may fail to activate upon injury, causing irreversible senescence. Chromatin experiences epigenetic drift, and DNA methylation changes affect lineage commitment and reduce transcriptional response to injury. The niche loses supportive cells; the ECM becomes stiffer. Reduced vascularization. The repair function is increasingly compromised
Tissue regeneration and wound repair processes	Scar-free regeneration prevails. Wounds heal rapidly, and complete restoration takes precedence over scar tissue formation, facilitated by high cellular plasticity, permissive extracellular matrix composition, high abundance of progenitor cells, and subdued inflammatory response and efficient remodeling. Structural and functional restoration is typically complete	Although repair remains highly efficient, a gradual transition toward the adult healing pattern begins. Regeneration continues to involve highly proliferative stem cells and efficient remodeling of the extracellular matrix. Scarring is usually limited, and tissue organization is almost completely restored	Repair follows the classic sequence of wound healing: hemostasis, inflammation, proliferation, and remodeling. The coordinated interaction among multiple cell types ensures the success of repair, the dynamic remodeling of the extracellular matrix, and local signaling	At this stage, tissue repair transitions toward a fibrotic phenotype. Inflammaging alters macrophage polarization and delays the resolution of inflammation; fibroblasts acquire a secretory phenotype associated with senescence; the extracellular matrix becomes stiffer; the stem cell niche loses its functionality. All these changes compromise regeneration and lead to fibrotic remodeling that interferes with function
Systemic integration and macro-level regulation of repair	A highly redundant, stage-specific somatotropic network is present (including placental HGH-V and CSH). This network maximizes tissue generation and safeguards early development independently of pituitary HGH.	The GH and IGF-I axis establishes the baseline for lifelong tissue repair capacity	Tissue repair is globally orchestrated by endocrine signals, neural inputs, and synchronized circadian rhythms. The glymphatic system executes efficient metabolic maintenance and waste clearance during SWS. The immune system maintains active surveillance and normal turnover of senescent cells	Multi-organ collapse and somatopause (dysregulation of the HGH/IGF-I axis) disrupt hormonal signaling and generate energetic deficits. Flattened circadian amplitudes and glymphatic system degradation severely compromise waste clearance. Immunosenescence, marked by thymic involution and senoinflammation, dismantles inflammatory resolution and senescent cell surveillance

Table 2 summarizes the primary mechanistic scales discussed in Sections 2–6. Sections 7, 8 are not listed as separate rows because they synthesize these mechanisms into an ontogenetic and clinical geroscience framework rather than introducing an additional biological scale.

During the fetal stage, stem cells are highly flexible; their chromatin is open, and they can proliferate rapidly. At this stage, their primary role is tissue construction rather than maintenance (Kobayashi and Kikyo, 2015). For example, fetal hematopoietic stem and progenitor cells form mixed-lineage colonies more readily than adult HSCs, reflecting a less specialized chromatin structure (Chen et al., 2019). This structure likely involves low H3K27me3 levels and a bivalent domain configuration, enabling rapid responses to changing developmental signals (Sachs et al., 2013; Voigt et al., 2013).

In childhood, stem cells appear to transition from building tissues to primarily maintaining them. This transition appears to occur mainly through the effects of sex hormones, Notch signaling, and proteins such as p57 and p27, which help stem cells enter the G_0_ resting state (Cho et al., 2019; Sato et al., 2026). At the same time, the stem cell microenvironment matures: the ECM changes, supportive cells become more specialized, and chromatin becomes more condensed, although regeneration-associated enhancers remain poised for activation (Adam et al., 2015; Gattazzo et al., 2014; Ugarte et al., 2015; Zamfirescu et al., 2022).

In adulthood, most stem cells remain quiescent, a state maintained by signals from Notch, FOXO3, p57, and mTOR suppression (Cheung and Rando, 2013; Liu and Sabatini, 2020; Urbán and Cheung, 2021). When tissue injury occurs, specific chromatin regions change, metabolism changes, and cells enter a G-alert state that prepares them to divide (Dong et al., 2022; Rumman et al., 2015; van Velthoven and Rando, 2019). Importantly, lineage memory is preserved through stable H3K27me3-mediated repression of non-lineage genes, meaning that plasticity at this stage is real but context-dependent (Osborne, 2026).

In old age, stem cell quiescence programs may fail to activate appropriately after injury. For example, in aging muscle, satellite cells can transition from a reversible resting state to a permanently inactive state because of elevated p16INK4a levels (Sousa-Victor, Gutarra, et al., 2014a; Sousa-Victor, Perdiguero, et al., 2014b). Chromatin also changes, with heterochromatin loss, reduced H3K9me3, and altered DNA methylation patterns that affect cell fate decisions and reduce responsiveness to damage (Lee et al., 2020). At the same time, the stem cell niche deteriorates because of ECM stiffening, reduced vascularization, and loss of supportive cells (de Mora and Juan, 2013). Senescent cells accumulate and produce chronic SASP signals that further suppress ASC function (Sun et al., 2014; Sutanto et al., 2025).

Overall, this pattern reflects a fundamental biological trade-off. The high flexibility of fetal stem cells is gradually replaced by adult stability and lineage commitment. The same systems that maintain this stability later become vulnerable points of failure during aging.

### 4.2 Lineage-specific exhaustion in somatic stem cell populations

While the overarching principles of niche degradation apply systemically, specific ASC populations exhibit distinct ontogenetic vulnerabilities. In the hematopoietic system, for instance, HSCs residing in the bone marrow undergo severe functional decline, even as their absolute numbers may increase with age. This decline is characterized by a distinct bias toward myeloid differentiation at the expense of lymphoid output, a transition that predisposes older adults to immune dysfunction and hematologic malignancies (Cui et al., 2025; Rix et al., 2022).

A parallel vulnerability is observed in the central nervous system. Neural stem cells (NSCs), localized in the subventricular and subgranular zones, are profoundly affected by the aged microenvironment. Chronic neuroinflammation, increased ECM stiffness, and reduced vascular support markedly impair NSC proliferation, directly correlating with age-related cognitive decline and memory impairment (Li and Han, 2025; Duve et al., 2025; Llorente et al., 2022).

This microenvironmental deterioration strongly influences the fate of barrier, vascular, and structural tissues. Endothelial stem cells (EnSCs), which are essential for vascular repair, are driven into senescence by elevated ROS and reduced nitric oxide (NO) bioavailability, significantly impairing vascular plasticity (Donato et al., 2015; Shimizu et al., 2023).

Similarly, in skeletal muscle, muscle stem cells (MuSCs) efficiently repair torn myofibers during youth. However, in old age, intrinsic mitochondrial dysfunction combined with disrupted fibro-adipogenic signaling in the aged niche skews MuSC differentiation toward a pro-fibrotic state, acting as a primary driver of sarcopenia (Libergoli and Almada, 2025; Uezumi et al., 2016).

Epithelial maintenance also falters dynamically across ontogeny. In the gut, LGR5+ intestinal stem cells (ISCs) maintain rapid epithelial turnover, but aging compromises their self-renewal through reduced canonical Wnt and Notch signaling from adjacent Paneth cells. This disruption increases susceptibility to gastrointestinal disorders, including inflammatory bowel disease (IBD), colorectal cancer, and age-related impairment of epithelial barrier integrity (Hou et al., 2021; Nalapareddy et al., 2022).

Concurrently, hair follicle stem cells (HFSCs) experience prolonged quiescence and dysregulated Wnt/β-catenin and BMP signaling. These changes lead to delayed cycling and age-related alopecia, even though the total HFSC numbers remain relatively stable (Jang et al., 2023; Peterson and Nair, 2022).

Finally, the reproductive system presents profound ontogenetic dimorphism. In males, human spermatogonial stem cells (SSCs) maintain lifelong spermatogenesis, although their surrounding testicular somatic niche undergoes age-related metabolic dysfunction that disrupts germ cell differentiation (Nie et al., 2022; Shamhari et al., 2023).

Conversely, the female reproductive system lacks an equivalent functional stem cell reservoir. Although the existence of adult oogonial stem cells (OSCs) was previously debated, high-resolution single-cell transcriptomics has conclusively refuted their presence in the adult human ovarian cortex, identifying the putative OSCs as perivascular cells (Wagner et al., 2020). Consequently, the female germline relies entirely on a finite fetal reserve of oocytes, making the ovary one of the earliest organs to undergo complete ontogenetic exhaustion.

Taken together, these lineage-specific and systemic observations underscore a central paradox of stem cell aging: despite retaining intrinsic self-renewal capacity, adult stem cells become functionally exhausted because the aged, inflammatory, and mechanically altered niche can no longer preserve quiescence, enforce balanced fate decisions, or support productive activation. As a result, self-renewal becomes insufficient to maintain tissue homeostasis.

### 4.3 Microenvironmental governance: the ontogenetic decline of the stem cell niche

The lifelong maintenance and regenerative capacity of ASCs are critically dependent on their microenvironment or “niche” (Schofield, 1978). Composed of supporting stromal cells, extracellular matrix (ECM), and vascular networks, the niche provides the biochemical and mechanical cues required to maintain ASCs in a reversible state of cell-cycle arrest known as quiescence (Fuchs et al., 2004; Gattazzo et al., 2014; Wagers, 2012).

Throughout the lifespan, the interplay between ASCs and their niche undergoes profound ontogenetic changes. In early life, the niche is highly permissive. Signaling pathways such as Wnt, Notch, and bone morphogenetic protein (BMP) are optimally balanced, allowing ASCs to rapidly exit quiescence, proliferate, and differentiate to build and repair tissues with high fidelity (Romito and Cobellis, 2016).

As the organism matures into adulthood, ASCs transition into self-renewing reservoirs. In this phase, they quietly replace cells lost to physiological turnover and mount localized proliferative responses to acute injury, carefully balancing self-renewal and differentiation (Urbán and Cheung, 2021).

Ultimately, as organisms enter senescence, stem cell function declines precipitously—a concept central to the “stem cell theory of aging” (Fukada et al., 2014). This decline is dual-faceted. Intrinsically, ASCs accumulate DNA damage, telomere attrition, and mitochondrial dysfunction, leading to replicative exhaustion or apoptosis (Ahmed et al., 2017; Picerno et al., 2021; Schultz and Sinclair, 2016). Extrinsically, the niche degrades: supporting cells produce fewer crucial growth factors, such as fibroblast growth factor (FGF) and epidermal growth factor (EGF), and dysregulated Wnt and Notch signaling skews stem cell fate toward senescence rather than self-renewal (Brunet and Rando, 2017).

Furthermore, the accumulation of senescent cells within the niche generates a SASP, exposing ASCs to chronic inflammaging-driven signals, including interleukin-6 (IL-6), tumor necrosis factor-**α** (TNF-**α**), and interleukin-1β (IL-1β) (Chandel et al., 2016; Conboy et al., 2005). This hostile, fibrotic environment suppresses ASC activation, delays wound healing, and drives age-related pathologies (Brunet et al., 2023; Liu et al., 2022).

In parallel, age-associated alterations in niche biomechanics—particularly increased extracellular matrix stiffness and aberrant integrin-mediated mechanotransduction—further constrain stem cell responsiveness. Mechanosensitive pathways, such as YAP/TAZ signaling, become chronically dysregulated in aged tissues, skewing lineage commitment and limiting regenerative plasticity. These findings indicate that biomechanical reconditioning may represent a therapeutic axis complementary to molecular rejuvenation.

### 4.4 The paradigm shift: from mesenchymal stem cells to medicinal signaling cells

Historically, mesenchymal stem cells (MSCs) were regarded as true multipotent progenitors capable of replacing diverse mesodermal tissues *in vivo*. However, recent evidence has driven a paradigm shift: in their native tissue environments, MSCs rarely exhibit canonical stem cell behaviors, such as sustained multilineage engraftment.

Originating from perivascular pericytes (Murray et al., 2014; da Silva et al., 2024), MSCs function primarily as damage sensors and immunomodulators. Recognizing this, Caplan (2017) proposed redefining the acronym as “Medicinal Signaling Cells.”

Rather than directly replacing tissue, MSCs regulate the perivascular niche by secreting a bioactive secretome composed of cytokines, miRNAs, and exosomes. Through the release of factors such as prostaglandin E2 (PGE2), indoleamine 2,3-dioxygenase (IDO), transforming growth factor-β (TGF-β), and interleukin-10 (IL-10), they suppress pathological inflammation and foster a pro-regenerative microenvironment (Song et al., 2020).

Furthermore, their ability to secrete antimicrobial peptides, such as cathelicidin (LL-37) and β-defensin 2, highlights their dynamic plasticity in responding to environmental threats (Alcayaga-Miranda et al., 2017; Krasnodembskaya et al., 2010). This immune-evasive and trophic profile forms the biological basis for their extensive use in allogeneic regenerative therapies (Rodríguez-Fuentes et al., 2021; Fernández-Garza et al., 2023).

### 4.5 Regulatory challenges of MSCs and the transition to cell-free secretome therapies

Given their direct engraftment potential and paracrine immunomodulatory properties, MSCs have been evaluated in thousands of clinical trials for diverse immune and inflammatory diseases. However, only a limited number of MSC-based products have been approved for clinical use by international regulatory agencies, and in the United States, only one has received FDA approval for the treatment of graft-versus-host disease (Blanc et al., 2025; Fernández-Garza et al., 2023). Several factors complicate the regulatory landscape of MSCs. First, clinical trial outcomes are highly heterogeneous because MSCs exhibit substantial donor-to-donor variability. Second, differences in MSC tissue sources, isolation procedures, and expansion methods further amplify this heterogeneity (Kukaj et al., 2025; Mattei et al., 2025). Although MSCs are generally considered safe and exhibit low immunogenicity, they remain cell-derived therapies and, therefore, must be manufactured under good manufacturing practice (GMP) requirements. This complicates scalability and requires strict documentation of safety, toxicity, oncogenicity, immunogenicity, efficacy outcomes, and adverse events in clinical trials (Lechanteur et al., 2021; Leonov et al., 2025).

One of the most promising strategies for addressing the translational and regulatory challenges of direct cell engraftment is to leverage the paracrine signaling properties of MSCs by using components of the MSC secretome collected from culture media. This secretome contains a diverse array of bioactive cytokines, microRNAs, extracellular vesicles (EVs), and exosomes (Giovannelli et al., 2023). EVs and exosomes can be isolated through sequential centrifugation and filtration and have been shown to deliver anti-inflammatory cytokines and microRNAs in multiple *in vitro* models. These findings have supported the development of genetically engineered exosomes designed to deliver upregulated microRNAs targeting specific disease pathways, including fibrosis, apoptosis, and immune modulation (De Sousa et al., 2023). Despite promising preclinical results, the therapeutic use of MSC-derived exosomes and EVs remains at an early stage. Multiple clinical trials are ongoing, but significant challenges persist, including donor-to-donor variability that is intrinsic to MSC-based therapies, scalability limitations, and the absence of fully standardized GMP requirements to ensure safety, quality, and long-term storage stability.

### 4.6 The paradigm shift in MSC therapy: from differentiation failure to efferocytosis-driven macrophage reprogramming

The concept of priming MSCs for differentiation to directly replace cells in injured tissue reveals a fundamental contradiction between their *ex vivo* potential and their actual *in vivo* function within the perivascular niche. For example, biophysical forces applied through specialized bioreactors or specific priming protocols can induce MSCs to enter defined differentiation pathways *in vitro* (Jahangir et al., 2020; Yeatts et al., 2013). However, translating this capacity into real clinical applications remains challenging. For instance, in cartilage tissue engineering, where MSCs have been used extensively, a major bottleneck is the tendency of primed MSCs to undergo hypertrophic differentiation after implantation rather than forming stable articular cartilage.

Furthermore, a significant immunological issue may complicate this approach. Although native MSCs exhibit low immunogenicity, forcing them toward differentiation through priming can upregulate major histocompatibility complex (MHC) class I and II surface markers, thus converting the otherwise immune-evasive cells into potential immunogenic targets for host T-cells (Ankrum et al., 2014; Lohan et al., 2014; Wang et al., 2019).

Although MSCs are considered immunoevasive, they can still be rapidly cleared after transplantation or injection. Interestingly, long-lasting therapeutic effects are often still observed, indicating that the MSCs’ mechanism of action may be indirect. A landmark study using a murine model of graft-versus-host disease (GvHD) demonstrated that MSCs are actively driven into caspase-dependent apoptosis by the recipient’s cytotoxic T-cells and natural killer (NK) cells shortly after infusion (Galleu et al., 2017). The authors then showed that host macrophages engulf apoptotic MSCs through efferocytosis, subsequently inducing macrophages to produce high levels of indoleamine 2,3-dioxygenase (IDO), a potent suppressor of exacerbated T-cell responses. Notably, when mice unable to phagocytose MSCs were used, the therapeutic effect of MSCs was largely absent.

Directly supporting this observation, a study by De Witte et al. (2018) showed that, after intravenous injection, MSCs become trapped in the lungs and lose membrane integrity. These dying cells are then phagocytosed by classical CD14^high^CD16^high^ monocytes and tissue-resident macrophages in the lungs and liver (Weiss and Dahlke, 2019). After engulfing MSCs, macrophages transition from a pro-inflammatory phenotype toward an anti-inflammatory M2-like state (Luque-Campos et al., 2021). These instructed macrophages also upregulate IL-10 and PD-L1, thereby attenuating the hyperinflammatory response (Weiss and Dahlke, 2019).

Although these mechanisms may explain why a therapeutic response persists even when MSCs are no longer present, they also raise new questions, including whether living cells are even required. Galleu et al. (2017) showed that the infusion of *ex vivo*-generated apoptotic MSCs was as effective as living MSCs in their models. This finding has initiated debate over whether future regulatory approvals should pivot toward manufacturing apoptotic bodies or cell ghosts rather than living cells. Because this mechanism relies on the patient’s own immune system to kill and engulf MSCs, patients with low baseline cytotoxic immune activity may fail to process MSCs properly, potentially leading to treatment failure (Galleu et al., 2017).

This raises a determinative question regarding patient selection: how can clinicians determine whether a patient has the specific immune profile required to unlock the therapeutic benefit of MSCs? This issue may also help explain why many MSC clinical trials have failed and why non-responders remain common (Caplan, 2018).

### 4.7 Contradictions, controversies, and unknowns in adult stem cell biology and their applications in regenerative medicine

Cell therapy and regenerative medicine often rely on assumptions about the biological traits of ASCs and progenitors, while overlooking factors that may influence outcomes, such as differentiation potency. The first assumption concerns stem cell homogeneity. Many studies have treated *ex vivo* ASCs, including bone marrow-derived MSCs, as uniform and predictable cell populations. However, multiple parameters define their biological characteristics, including tissue source, donor age, health status, and culture conditions. These variables determine specific cellular properties, such as proliferation, differentiation potential, and secretome composition. The second assumption is that epigenetic age directly controls functionality. The field often assumes that resetting a cell’s epigenetic clock leads to genuine functional rejuvenation. This view presumes that DNA methylation profiles are the primary cause of biological aging rather than downstream biomarkers (Bell et al., 2019). Finally, the assumption that stem cell plasticity is linear tends to overestimate the capacity of ASCs to transdifferentiate or dedifferentiate into unrelated cell lineages under certain conditions, such as stress (Strauss et al., 2012).

#### 4.7.1 The identity and nomenclature controversy of MSCs

Arnold Caplan named these cells “mesenchymal stem cells” based on observations that they could differentiate into multiple mesenchymal lineages *in vitro*, such as bone and cartilage (Caplan, 1991). The International Society for Cell and Gene Therapy (ISCT) later suggested that the acronym “MSC” should preferentially refer to “mesenchymal stromal cells” rather than “mesenchymal stem cells” unless proof of *in vivo* self-renewal and differentiation is provided (Viswanathan et al., 2019). Native MSCs have repeatedly been shown to function primarily as perivascular support cells within the stem cell niche, rather than as a direct source of newly differentiated replacement cells (Caplan, 2017; Crippa and Bernardo, 2018; Feng et al., 2010). Nevertheless, numerous studies still assume that these cells behave as stem cells after implantation, thereby mischaracterizing therapeutic effects that largely arise from transient paracrine activity.

#### 4.7.2 Mechanism of action: true differentiation vs. paracrine signaling

For many years, it was widely assumed that transplanted ASCs could home to damaged tissues or organs, engraft, and replace lost cells by differentiating *in situ*. Recent evidence now shows that this phenomenon strongly depends on the specific cell type, delivery route, and target tissue and, therefore, cannot be generalized as a universal mechanism. Systemically or locally delivered MSCs do not permanently engraft or differentiate within target tissues; in most cases, they are rapidly cleared by the body. Their therapeutic effects largely depend on paracrine signaling, through which cells secrete cytokines, chemokines, and extracellular vesicles that modulate multiple signaling pathways (Chang et al., 2021; Hocking and Gibran, 2010). For this reason, particularly in the case of MSCs, Arnold Caplan repeatedly advocated renaming them as “medicinal signaling cells” (Caplan, 2017). Further work is still needed to elucidate the precise molecular composition of this secretome, a task made difficult by the highly dynamic capacity of these cells to adapt to changes in their microenvironment.

In contrast to MSCs, HSCs possess the capacity to engraft and differentiate *in situ* within the bone marrow, their target organ (Wilton and Hasserjian, 2023). HSCs express high levels of CXCR4, which allows them to follow the CXCL12 gradient generated by abundant reticular cells (CAR) in the bone marrow (Ratajczak et al., 2013).

Transplanted HSCs follow this gradient and migrate directly to the bone marrow, crossing the endothelial wall of bone marrow sinusoids through an active multistep adhesion cascade involving integrins, such as VLA-4, and selectins (Sahin and Buitenhuis, 2012). HSCs can then re-establish hematopoiesis by differentiating in a hierarchical, multilineage fashion to generate billions of functional red blood cells, platelets, and immune cells (Dong et al., 2020).

Transplanted muscle stem cells can also engraft and differentiate *in situ*. However, unlike HSCs, they lack robust homing machinery for injured muscle and, therefore, usually require direct delivery to the injured tissue (Boyer et al., 2021).

#### 4.7.3 ASC plasticity and epigenetic status

For several decades, particularly during the late 1990s and early 2000s, numerous groups reported that certain ASCs, such as MSCs and HSCs, could transdifferentiate into multiple lineages outside their germ-layer restrictions. These transdifferentiation and dedifferentiation abilities may have been overestimated (Hombach-Klonisch et al., 2008; Horwitz, 2003; Morshead et al., 2002; Wells, 2002). Robust lineage-tracing experiments eventually indicated that many of these observations were likely the result of cell fusion events or contamination in heterogeneous cell cultures rather than true reprogramming (Hombach-Klonisch et al., 2008; Liu and Rao, 2003; Vassilopoulos and Russell, 2003; Wang et al., 2019). Although ASCs cannot readily cross germ-layer barriers under normal conditions, their epigenetic signatures are not entirely fixed; the microenvironment can influence chromatin accessibility and, in some contexts, trigger transition in cell identity. When cells are exposed to stressors or other external factors, they may enter a state of partial dedifferentiation (Blum, 2015; Jadhav et al., 2017; Krstic et al., 2017; Tataranu and Rizea, 2025). However, it remains unclear how this process can be manipulated for therapeutic utility. In this context, cells forced to transdifferentiate or dedifferentiate retain strong epigenetic memory of their tissue of origin (Hörmanseder, 2021; Scott et al., 2023), making it difficult for them to maintain a stable transdifferentiated state (Cho and Ryoo, 2018). Consequently, much of the observed plasticity may be an artifact of *in vitro* culture conditions, which constitute a highly stressful environment (Kim & Kino-oka, 2026; Somoza et al., 2008; Wells, 2002). Therefore, it remains highly controversial whether ASCs can truly display stable transdifferentiation or dedifferentiation states under normal physiological conditions.

## 5 Tissue-level regeneration and the wound healing paradigm

While cellular repair focuses on the plasticity and replacement of individual cells, tissue repair represents a higher-order, multicellular orchestration. Rebuilding functional tissue requires the synchronized interaction of diverse cell lineages, dynamic ECM remodeling, and localized mechanical signaling. This process first manifests as a generalized wound-healing response and is then further specialized according to the unique architectural demands of specific organs.

### 5.1 The wound repair continuum

**FIGURE 3:**
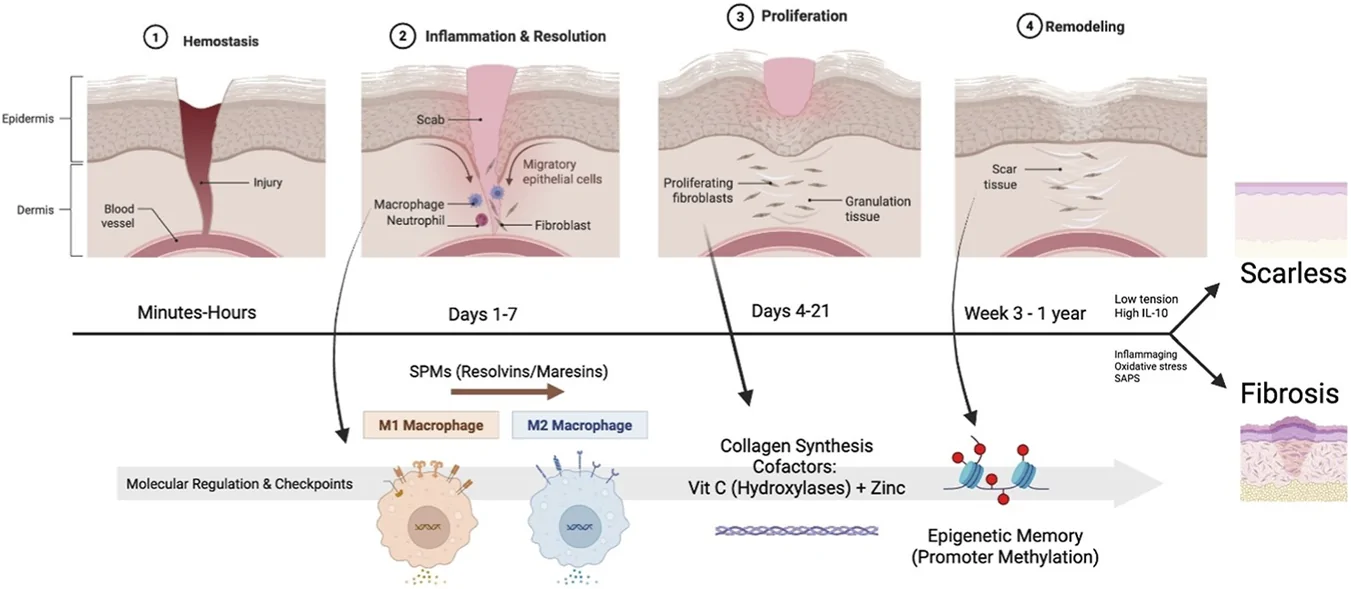
Reparative continuum: from cellular orchestration to molecular regulation. Schematic representation of the four overlapping phases of tissue repair. The figure highlights the M1-to-M2 macrophage phenotypic transition mediated by specialized pro-resolving mediators (SPMs) and the dependence on nutritional cofactors, including vitamin C and zinc, for enzymatic matrix remodeling. It further illustrates the ontogenetic bifurcation of regenerative outcomes: fetal scarless regeneration *versus* adult fibrosis driven by inflammaging and oxidative stress.

While homeostatic tissue renewal manages daily cellular turnover, acute tissue injury triggers a highly orchestrated, evolutionarily conserved survival mechanism: the wound-healing response. Across the human lifespan, this response invariably progresses through four overlapping phases (Figure 3).

#### 5.1.1 Hemostasis, innate immune recruitment, and inflammatory resolution

The body’s immediate response to acute injury focuses on achieving hemostasis within minutes to hours. This phase begins when blood escapes into the wound, triggering the extrinsic coagulation pathway and releasing substances such as serotonin to induce localized vasoconstriction (Reinke and Sorg, 2012). Platelets activate upon contact with exposed subendothelial collagen, forming a hemostatic plug and degranulating to release growth factors and cytokines, including C-X-C motif chemokine ligand 8 (CXCL8), interleukin-1⍺ (IL-1⍺), IL-1β, IL-6, and TNF-⍺ (Björk et al., 1985; Palta et al., 2014). Crucially, this platelet activity establishes an initial provisional matrix of fibronectin, vitronectin, and thrombospondins, creating a structural scaffold for subsequent cell migration (King et al., 2014).

Simultaneously, complement activation triggers histamine release, promoting capillary dilation and vascular permeability and marking the transition into the inflammatory stage (Sinno and Prakash, 2013). This innate immune response is initiated by DAMPs and pathogen-associated molecular patterns (PAMPs), which activate pattern-recognition receptors on resident macrophages and mast cells (Chen et al., 2017; Cook-Mills and Deem, 2005). The resulting signaling cascade, largely driven by NF-κB, facilitates the extravasation of circulating leukocytes (Vestweber, 2015). Neutrophils arrive first, clearing necrotic tissue and pathogens through phagocytosis, ROS generation, and the deployment of neutrophil extracellular traps (NETs) *(* Brinkmann et al., 2004; Kolaczkowska and Kubes, 2013; Segel et al., 2011).

Following neutrophil influx, circulating monocytes migrate into the wound and differentiate into macrophages. Macrophages serve as the primary conductors of the reparative response. Initially, they adopt a classically activated, pro-inflammatory M1-like phenotype under the influence of lipopolysaccharide (LPS) and interferon-*γ* (IFN-*γ*), producing ROS and clearing apoptotic neutrophils through efferocytosis (Delavary et al., 2011; Mantovani and Locati, 2005).

However, for healing to progress, these macrophages must undergo a phenotypic switch toward an alternatively activated, pro-resolving (M2-like) state. These pro-repair macrophages secrete anti-inflammatory cytokines, including interleukin-4 (IL-4), IL-10, interleukin-13 (IL-13), and arginase, thereby driving wound closure and new blood-vessel formation (Campbell et al., 2013; Jetten et al., 2014).

Selective ablation experiments confirm that failure to execute this macrophage transition causes uncontrolled inflammation, delayed wound closure, and severe tissue damage (Lucas et al., 2010; Mirza et al., 2009). This transition is fundamentally governed by the Janus kinase-signal transducer and activator of transcription (JAK–STAT) pathway, which serves as the primary intracellular conduit for the cytokines mentioned above. Specifically, while IFN-*γ* and IL-6 typically activate STAT1 and STAT3 to reinforce M1-like proinflammatory programs, IL-4 and IL-13 signal through STAT6 to drive the M2-like reparative phenotype. This signaling axis acts as a molecular rheostat, orchestrating the resolution of inflammation and the initiation of tissue remodeling (Hu et al., 2021; Villarino et al., 2017).

#### 5.1.2 Proliferation, angiogenesis, and re-epithelialization

As inflammation resolves, the proliferative phase begins, characterized by angiogenesis, granulation tissue formation, and re-epithelialization (Ellis et al., 2018). Pro-repair macrophages secrete vascular endothelial growth factor (VEGF), which is essential for the sprouting and anastomosis of new capillaries (Brancato and Albina, 2011; Fantin et al., 2010). Concurrently, fibroblasts migrate into the wound, stimulated by macrophage-derived platelet-derived growth factor-BB (PDGF-BB), TNF-⍺, IL-1β, and IL-6 (Brauchle et al., 1994; Takehara, 2000). These fibroblasts synthesize type III collagen, forming the bulk of the granulation tissue.

Above this reforming stroma, epithelial stem cells from the basal layer and hair follicle root sheath proliferate and migrate to restore the epidermal barrier, a process driven by EGF, keratinocyte growth factor (KGF), and transforming growth factor-⍺ (TGF-⍺) (Lau et al., 2009; Schultz et al., 1991).

#### 5.1.3 Tissue remodeling and epigenetic memory

The final stage, remodeling, begins weeks after injury and can persist for more than a year. During this phase, provisional type III collagen is gradually replaced by stronger type I collagen, ultimately restoring up to 80% of the tissue’s original tensile strength. This structural reorganization is executed by myofibroblasts—specialized contractile cells that differentiate from fibroblasts in response to mechanical stress and TGF-β signaling. Myofibroblasts express ⍺-smooth muscle actin (⍺-SMA) to draw wound edges together and secrete matrix metalloproteinases (MMPs) to reorganize the matrix (Darby et al., 2014; Yannas et al., 2017).

Crucially, persistent myofibroblast activation is driven by an epigenetic “mechanical memory” that locks these cells into a profibrotic state. This process begins at the level of DNA methylation, where active demethylation of the *ACTA2* promoter is strictly required to initiate myofibroblast differentiation (Hu et al., 2010).

Once activated, these cells translate the physical stiffness of the surrounding extracellular matrix into lasting chromatin alterations. Specifically, mechanosensitive chromatin-remodeling enzymes, including histone acetyltransferases (HATs), increase the transcriptional accessibility of profibrotic genes (Kureel et al., 2025; Qiu et al., 2025).

Together, this dynamic epigenetic plasticity allows fibroblasts to retain long-term molecular memory of the mechanical environment, driving pathological hypertrophic scarring even after the initial physical tension has resolved.

#### 5.1.4 Extrinsic modulation: nutritional cofactors and metabolic dysregulation

The efficiency of these intrinsic cellular mechanisms is strongly modulated by systemic and environmental factors. Adequate nutritional cofactors are indispensable; for instance, vitamin C is an essential cofactor for prolyl-4-hydroxylase, without which collagen triple helices remain unstable and rapidly degrade (Pullar et al., 2017).

Similarly, zinc is a structural requirement for MMPs involved in ECM remodeling (Lin et al., 2018). Conversely, pathological metabolic states, such as chronic hyperglycemia in diabetes, disrupt lipid metabolism and induce excessive oxidative stress, impairing fibroblast proliferation, delaying macrophage transition, and severely compromising angiogenesis, as evidenced by ulcers and ineffective healing in patients with diabetes (Wang et al., 2024).

Emerging evidence further highlights metabolic flexibility as a determinant of reparative efficiency. Effective wound healing requires coordinated metabolic switching between glycolysis and oxidative phosphorylation in immune cells and fibroblasts. Aging disrupts this adaptive metabolic reprogramming, thus prolonging inflammatory phases and impairing matrix remodeling. Thus, metabolic dysfunction may represent a mechanistic bridge linking systemic aging hallmarks to localized fibrotic outcomes.

### 5.2 Organ-specific tissue repair dynamics

Beyond general wound healing, tissue repair mechanisms are highly specialized and governed by the distinct cellular turnover rates and stem cell niches of individual organ systems.

#### 5.2.1 The central nervous system: neuroplasticity vs. neurodegeneration

The human brain exhibits profound ontogenetic changes in repair capacity. During childhood and adolescence, the brain is characterized by extensive neuroplasticity and active neurogenesis, primarily localized to the SVZ of the lateral ventricles and the SGZ of the hippocampus (Dennis et al., 2016).

This structural reorganization is sustained by dynamic proteostasis, robust brain-derived neurotrophic factor (BDNF) and nerve growth factor (NGF) signaling, and highly efficient mitochondrial control of dendritic remodeling. However, this capacity declines precipitously with age.

At the cellular level, this decline is driven by the collapse of intrinsic repair networks and pathological changes in glial cell function. Astrocytes lose their ability to maintain the blood–brain barrier (BBB), while microglia adopt a persistent, senescence-associated proinflammatory phenotype that exacerbates neuronal apoptosis and drives age-related cognitive decline (Guo et al., 2022; Palmer and Ousman, 2018).

#### 5.2.2 Heart: myocardial regeneration and fibrosis

The myocardium provides one of the most striking examples of restricted ontogenetic plasticity. During embryonic and early neonatal stages, the mammalian heart possesses a remarkable capacity to replace damaged cardiomyocytes and undergo functional remodeling with *restitutio ad integrum* (Porrello et al., 2011). At the molecular level, this plasticity is governed by the Hippo/Yes-associated protein (YAP) signaling pathway, which maintains cardiomyocytes in a proliferative state, and by the activation of the neuregulin-1 (NRG1)/Erb-b2 receptor tyrosine kinase 4 (ErbB4) axis (Wang et al., 2023).

This regenerative capacity is rapidly silenced shortly after birth as the Hippo/YAP and NRG1/ErbB4 pathways are downregulated. In adults, the number of functional cardiac stem cells (CSCs) declines exponentially. Consequently, adult cardiac repair relies on fibrotic scarring rather than cellular replacement, leading to progressive architectural changes that restrict the efficiency of blood ejection and eventually lead to heart failure (Guo et al., 2022).

#### 5.2.3 Liver: regenerative robustness and exhaustion

Unlike the heart or the brain, the human liver retains an extraordinary, evolutionarily conserved regenerative capacity throughout adulthood. Following acute injury or partial resection, the liver can regenerate its mass through the coordinated proliferation of existing cell lineages. This process is critically orchestrated by the innate immune system; hepatic macrophages, known as Kupffer cells, secrete cytokines such as IL-6, which activate the STAT3 signaling pathway (Forbes and Newsome, 2016). Simultaneously, the Hippo/YAP signaling pathway acts as a crucial rheostat for liver size and regeneration.

However, this resilience is not infinite. In contexts of severe chronic injury, repetitive DNA damage, and continuous inflammatory signaling, repair mechanisms become overwhelmed. This drives hepatic stellate cells to deposit excessive collagen, transitioning the trajectory from functional regeneration toward established cirrhosis, an advanced fibrotic state that is largely beyond repair.

## 6 Systemic integration and macro-level regulation of repair

Although local cellular networks and epigenetic modifications govern the immediate response to injury, tissue regeneration does not occur in a physiological vacuum. Rather, it is orchestrated by higher-order systemic cues, including endocrine signaling, neural inputs, and circadian rhythms. This macro-level regulation ensures that local repair processes are metabolically supported and synchronized with the organism’s overall physiological state. Hormones act as central nodes in this network; for instance, insulin, insulin-like growth factor 1 (IGF-I), and estrogens profoundly enhance cell proliferation and extracellular matrix synthesis, thereby shaping regenerative outcomes in tissues such as the intestinal epithelium, skeletal muscle, and skin (Kotsifaki et al., 2024; Li X. et al., 2024). Concurrently, circadian rhythms provide a global temporal framework. With nearly 40% of protein-coding genes under circadian control in at least one organ, regenerative processes across multiple niches are intrinsically synchronized with daily environmental and metabolic cycles (Paatela et al., 2019).

### 6.1 The ontogeny of the somatotropic axis: from fetal redundancy to somatopause

The role of systemic growth factors in defining repair and developmental capacity across ontogeny is strongly anchored by the growth hormone (GH)/IGF-I axis (Fernández-Garza et al., 2025). Historically, human growth hormone (HGH) was thought to exert minimal influence before birth, because infants later diagnosed with hypothalamic GH deficiency (GHD) often exhibit normal growth during their first year (Parks, 2004). However, modern evidence reveals a highly redundant and stage-specific somatotropic network that safeguards early development and establishes the baseline for lifelong tissue repair capacity.

During intrauterine development, IGF-I serves as an indispensable driver of tissue generation, particularly during the second and third trimesters. Clinical observations of complete *IGF-I* gene deletions, which lead to severe intrauterine growth restriction, underscore its absolute necessity (Woods et al., 1996). Importantly, fetal IGF-I production can occur independently of pituitary HGH, indicating an evolutionary redundancy designed to protect the developing organism from localized hormonal deficiencies.

This systemic redundancy is further exemplified by genetic disruptions of the HGH/IGF-I axis. Conditions such as Laron syndrome, caused by HGH receptor (GHR) mutations, lead to variable postnatal growth insensitivity but relatively mild prenatal phenotypes (Hwa et al., 2021). This prenatal protection is orchestrated by compensatory actions of placental-derived hormones. After the first trimester, maternal pituitary HGH (GH-N) is superseded by the placental HGH variant (HGH-V) and chorionic somatomammotropin hormone (CSH). Secreted continuously into the maternal circulation by the syncytiotrophoblast, HGH-V drives maternal hepatic IGF-I production and induces transient insulin resistance, thus markedly increasing nutrient availability for fetal tissue building (Barrera-Saldaña, 1998; Lacroix et al., 2002; Newbern and Freemark, 2011).

The importance of this intricate crosstalk is highlighted by cases involving mutant HGH variants, such as Arg77Cys, or homozygous deletions of *hGH-V* and *hCSH* genes, which dismantle this redundant network and precipitate profound growth retardation (Takahashi et al., 1996; Rygaard et al., 1998). Ultimately, the HGH/IGF-I axis, which is robust and highly redundant during early ontogeny to maximize tissue generation, is progressively dysregulated during adulthood. This somatopause contributes substantially to the systemic decline in repair capacity observed with aging (Bartke, 2019).

### 6.2 Neuro-reparative mechanisms: the glymphatic system and circadian rhythms

In parallel with endocrine regulation, the central nervous system utilizes a highly specialized and deeply interconnected systemic mechanism for maintenance and repair: the synchronization of circadian molecular clocks with the glymphatic clearance system (Stowe and McClung, 2023; Dragoi et al., 2025). At the cellular level, the circadian network is governed by the suprachiasmatic nucleus—the “master clock”—which synchronizes peripheral clocks in virtually every cell. This biological rhythm is driven by a genetically programmed transcriptional–translational feedback loop (TTFL), in which core clock proteins such as BMAL1 and CLOCK activate repressor genes, including Period (*PER*) and Cryptochrome (*CRY*), which subsequently inhibit the complex and establish a 24-h oscillation (Stowe and McClung, 2023; Verma et al., 2023). This molecular timekeeping is indispensable for cellular repair, particularly in regulating redox homeostasis. The clock directly dictates the expression of the antioxidant transcription factor NRF2, a downstream target of brain and muscle Arnt-like protein 1 (BMAL1). Consequently, chronodisruption immediately compromises cellular antioxidant defenses, leaving tissues vulnerable to oxidative stress.

While the molecular clock manages intracellular defense, the glymphatic system executes macro-scale metabolic maintenance. Lacking conventional lymphatic architecture, the brain relies on this glial-dependent pathway for dynamic exchange between cerebrospinal fluid (CSF) and interstitial fluid (ISF) (Benveniste et al., 2018; Voumvourakis et al., 2023). CSF enters the brain parenchyma along the periarterial Virchow–Robin spaces, a convective influx strongly facilitated by aquaporin-4 (AQP4) water channels polarized at astrocytic end-feet (Benveniste et al., 2018; Xiong et al., 2024). This fluid traverses the interstitium, collecting soluble waste and metabolic byproducts of neuronal activity, before exiting through perivenular spaces toward the cervical lymphatic network (Kipnis et al., 2025).

Crucially, the efficacy of the glymphatic system is closely linked to the circadian sleep–wake cycle. Glymphatic convective flow is markedly suppressed during wakefulness and surges during sleep, particularly during slow-wave sleep (SWS), driven by physiological expansion of the interstitial space that facilitates optimal CSF–ISF exchange (Benveniste et al., 2018; Voumvourakis et al., 2023; Xiong et al., 2024). Furthermore, this clearance pathway serves as a neuroimmune bridge: immune cells residing in brain-border niches, such as the meninges, monitor solute efflux and modulate fluid flow, integrating waste removal with active immune surveillance (Kipnis et al., 2025).

The ontogenetic trajectory of these neuro-reparative systems culminates in a precipitous age-related decline. Advancing age is characterized by diminished melatonin secretion, flattened circadian amplitudes, and a marked reduction in SWS (Stowe and McClung, 2023; Verma et al., 2023). Concurrently, the glymphatic system undergoes physical degradation, specifically the loss of AQP4 polarization at astrocytic end-feet (Benveniste et al., 2018; Xiong et al., 2024). This dual collapse of molecular timekeeping and physical clearance mechanisms critically impairs the brain’s ability to clear neurotoxic proteins, such as amyloid-beta and tau, establishing a pathophysiological foundation for age-related neurodegenerative diseases. Beyond circadian disruption, inter-organ communication through circulating extracellular vesicles and endocrine-derived microRNAs is increasingly recognized as a systemic integrator of repair responses. Age-related decline in the coherence of these signaling networks may compromise the synchronization of tissue-level regeneration, indicating that regenerative failure partly reflects a breakdown in organismal communication fidelity.

### 6.3 Systemic senescence: multi-organ collapse and immunosenescence

The localized failure of tissue-specific repair in older organisms is compounded by a global systemic collapse spanning multiple organ networks. Within the endocrine system, widespread atrophy profoundly disrupts hormonal signaling. The anterior pituitary reduces HGH secretion, the thyroid undergoes follicular atrophy and impaired peroxidase activity, and the adrenal cortex develops fibrotic remodeling. Together, these changes reduce the production of key anabolic hormones, including dehydroepiandrosterone (DHEA).

Concurrently, the endocrine pancreas exhibits β-cell depletion and interstitial fibrosis, severely compromising metabolic homeostasis and depleting the energetic substrates required for whole-body tissue repair (Beld et al., 2018).

This energetic deficit is further exacerbated by digestive deterioration. As LGR5+ stem cell renewal declines, the gastrointestinal mucosa thins. Subsequent collagen deposition and loss of intestinal wall elasticity reduce nutrient absorption and compromise the gut immune barrier, effectively depriving the aging organism of the nutritional cofactors essential for cellular maintenance (Dumic et al., 2019). This systemic vulnerability is further magnified by immunosenescence, which dismantles both defense and repair coordination.

Immunosenescence is caused by gradual remodeling and functional decline of the immune system with aging. It is marked by reduced capacity to respond to infections and vaccines and by increased susceptibility to age-related diseases (Liu et al., 2023). This process involves dysregulation of both innate and adaptive immunity and is characterized by thymic involution, inflammaging, and the accumulation of senescent cells (SnCs) (Gong et al., 2025).

Thymic involution leads to reduced production of naïve T-cells, accumulation of late-differentiated memory T-cells in the periphery, and impaired migration of newly generated T-cells (Flores et al., 1999; Lynch et al., 2009).

The peripheral naïve T-cell pool shrinks, while exhausted memory T-cells accumulate and chronically secrete proinflammatory cytokines such as IFN-*γ* and TNF-⍺, thereby sustaining systemic inflammaging (Oh et al., 2019). Coupled with age-related metabolic alterations in HSCs and functional decline in macrophages, this immune deterioration actively disrupts the inflammatory resolution phases required for efficient wound healing and functional tissue regeneration (Proshkina et al., 2020; Guo et al., 2022).

#### 6.3.1 Changes in innate immunity

Natural killer (NK) cells also undergo significant age-related changes. Although their overall numbers may increase, the proportion of functionally active CD56^bright^ subsets declines (Campos et al., 2015). Aging is associated with reduced expression of activating receptors and increased expression of inhibitory receptors, such as killer cell immunoglobulin-like receptors (KIRs). Functionally, aged NK cells display impaired degranulation and reduced perforin release, potentially because of defects in Ca^2+^-dependent exocytosis regulated by proteins such as Munc13-4 (Bin et al., 2018). Overall, NK cell aging is characterized by impaired maturation, reduced cytotoxicity, and a transition toward a proinflammatory phenotype.

Dendritic cells (DCs), key regulators of antigen presentation and T-cell activation, are also compromised with age. Aged DCs exhibit reduced expression of major histocompatibility complex (MHC) molecules and costimulatory molecules such as CD40, leading to impaired cytokine production in response to stimuli such as LPS and a diminished capacity to activate CD4^+^ T-cells (Pereira et al., 2011). In addition, mitochondrial dysfunction in aged DCs contributes to reduced energy production and increased oxidative stress, further impairing their immunological function (Chougnet et al., 2015).

#### 6.3.2 Changes in adaptive immunity

The lymphoid compartment is also significantly affected by aging. Although both innate lymphoid cells, such as NK cells, and adaptive immune cells, including T and B lymphocytes, are affected, the most pronounced changes occur within adaptive immunity. Aging is associated with reduced production and impaired function of naïve T- and B-cells, accompanied by the expansion of memory cell populations (Lazuardi et al., 2005).

At the cellular level, senescent T-cells undergo significant phenotypic and functional changes, including downregulation of costimulatory molecules such as CD27 and CD28 and upregulation of senescence-associated markers, including killer cell lectin-like receptor subfamily G member 1 (KLRG1) and CD57 (Chen et al., 2026).

In addition, aging induces increased expression of immune checkpoint molecules such as LAG-3, PD-1, and CTLA-4, further contributing to T-cell dysfunction (Li et al., 2026; Lukas Yani et al., 2018). Functionally, senescent T-cells exhibit reduced cytotoxic capacity, with diminished production of key effector molecules such as IFN-γ, granzyme B, and perforin (Li et al., 2026; Yang et al., 2005).

B-cells similarly exhibit age-associated dysfunction, including defects in antibody production and impaired immunoglobulin class switching (Frasca et al., 2004; 2008). During infections, this translates into reduced B-cell frequencies, weaker antibody responses, and diminished protective immunity, ultimately compromising vaccine efficacy in older populations. Despite this decline, aging is often associated with an increased proportion of memory T- and B-cells, particularly memory CD8^+^ T-cells (Pulko et al., 2016; Vescovini et al., 2014).

#### 6.3.3 Systemic consequences: inflammaging and senoinflammation

Aged myeloid cells are characterized by elevated secretion of proinflammatory cytokines such as TNF-α and IL-6, contributing to a chronic low-grade inflammatory state termed “senoinflammation” (Hearps et al., 2012; Verschoor et al., 2013). Neutrophils, typically short-lived innate immune cells, exhibit extended lifespan and abnormal morphological features during aging, including hypersegmentation in secondary lymphoid organs and bone marrow. These aged neutrophils remain responsive to stimuli such as LPS, producing excessive cytokines, while also displaying dysregulated recruitment due to altered chemokine signaling and inflammatory responses.

Similarly, aging promotes proinflammatory polarization of monocytes, partly driven by increased levels of circulating saturated fatty acids. This change leads to enhanced production of IL-6 and TNF-α alongside reduced secretion of anti-inflammatory mediators such as IL-10 and transforming growth factor-β1 (TGF-β1) (Pararasa et al., 2016).

In parallel, macrophages and monocytes exhibit a marked decline in phagocytic capacity, leading to the accumulation of cellular debris, sustained sterile inflammation, and exacerbation of tissue damage and aging (Moss et al., 2024).

### 6.4 Immune surveillance of senescent cells

Components of the SASP serve as signals for immune-mediated clearance of senescent cells. In the innate immune system, NK cells are key mediators of senescence immunosurveillance. SASP factors recruit NK cells, which recognize stress-induced ligands on senescent cells through the activating receptor NKG2D. Engagement of NKG2D with ligands such as MHC class I chain-related protein A (MICA) and UL16-binding protein 2 (ULBP2) triggers NK-cell cytotoxicity through granule exocytosis, leading to elimination of senescent cells (Ovadya et al., 2018; Saghiv et al., 2017).

Macrophages also play a central role in this process as essential components of the myeloid lineage, capable of selectively phagocytosing damaged, apoptotic, and senescent cells. The cyclin-dependent kinase inhibitor p21 contributes to shaping the senescent secretory phenotype through retinoblastoma (RB)-dependent transcriptional programs, regulating the secretion of cytokines, chemokines, and extracellular matrix components. Among these, CXCL14 promotes rapid recruitment of macrophages to p21-positive senescent cells. When p21 expression is sustained, macrophages can polarize toward a proinflammatory phenotype, further promoting the recruitment of cytotoxic CD8^+^ T-lymphocytes to facilitate senescent cell clearance (Levi et al., 2026; Papismadov et al., 2024; Sturmlechner et al., 2021).

Neutrophils, as early responders of the innate immune system, also contribute to senescence surveillance. They can detect oncogenic stress during early tumorigenesis and initiate immune responses against transformed cells. For instance, in diabetic retinopathy, senescent endothelial cells release proinflammatory signals that recruit neutrophils (Debiesse et al., 2025). These neutrophils form neutrophil extracellular traps (NETs), which mediate apoptosis and clearance of senescent vascular cells, thereby reducing pathological angiogenesis and supporting tissue repair (Binet et al., 2020).

In addition to innate immunity, adaptive immune responses are increasingly recognized as critical regulators of senescent cell clearance. CD4^+^ T-cells can acquire cytotoxic functions in senescence-rich environments. For example, interactions between RAS-specific CD4^+^ T-cells and recruited monocytes promote senescence-mediated antitumor immunity, including clearance of senescent hepatocytes and suppression of hepatocellular carcinoma development (Czyzyk et al., 2003).

Similarly, senescent fibroblasts expressing HLA class II and viral antigens, such as HCMV glycoprotein B, can be targeted and eliminated by cytotoxic CD4^+^ T-lymphocytes (Hasegawa et al., 2023). The transcription factor EOMES drives the differentiation of these lymphocytes into cytotoxic effectors, highlighting their role in regulating senescent cell burden during aging and chronic inflammation (Elyahu et al., 2024).

CD8^+^ T-cells also play a pivotal role in senescence immunosurveillance, particularly in aging and cancer. Senescent cells can activate CD8^+^ T-cell responses through the secretion of proinflammatory cytokines and enhanced antigen presentation. Type I interferon signaling upregulates antigen-processing machinery and MHC class I expression in senescent cells, facilitating recognition by T-cell receptors (TCRs) and promoting cytotoxic elimination (Wang et al., 2022).

Finally, humoral immunity may also contribute to senescent cell clearance. Immunization studies have shown that senescent cells can induce antibody production, including antibodies targeting oxidized vimentin, a surface marker of senescent cells (Frescas et al., 2017). These findings indicate that B-cells may participate in senescence surveillance through antibody-mediated mechanisms, although this function may decline with age, contributing to the accumulation of senescent cells.

Overall, the immune system plays a central role in clearing senescent cells. However, senescent cells can utilize multiple strategies to evade immune surveillance, including direct cell–cell interactions and inflammation-driven mechanisms. This evasion is further exacerbated during aging, when immune surveillance becomes progressively impaired. An important emerging area of research is determining whether age-associated immune dysfunction drives the accumulation of senescent cells or whether increasing senescent-cell burden further deteriorates immune function.

## 7 Synthesizing the ontogenetic continuum: from embryonic plasticity to senescent decline

Integrating the genomic, intracellular, cellular, tissue, and systemic scales of repair reveals a central physiological principle: the organism’s life stage is a major determinant of reparative success. The transition from embryonic plasticity to senescent decline determines whether tissue repair proceeds toward seamless regeneration or fibrotic scarring.

### 7.1 The ontogenetic bifurcation: scarless fetal healing vs. age-related fibrosis

The most profound demonstration of ontogenetic influence on tissue repair lies in the stark contrast between early embryonic and senescent wound healing. Mammalian embryos exhibit a unique capacity for scarless wound healing. This *restitutio ad integrum* is facilitated by an ECM heavily enriched in hyaluronic acid and type III collagen, alongside an altered mechanical environment characterized by lower contractile forces (Moore et al., 2018; Walmsley et al., 2015).

Conversely, aging transforms the reparative landscape into a profibrotic environment. The aging niche is characterized by inflammaging that alters macrophage polarization, prevents the critical M1-to-M2 switch, and delays the resolution of inflammation (Kushioka et al., 2023; Sica et al., 2015).

Aged fibroblasts adopt a SASP-like phenotype and release tissue-degrading enzymes, while structural ECM proteins become progressively stiffer through cross-linking and glycosylation (Kular et al., 2014). Furthermore, aging impairs the functional output of localized stem cell niches, such as HFSCs, which undergo prolonged quiescence because of dysregulated Wnt/β-catenin and BMP signaling (Jang et al., 2023).

Ultimately, this age-associated dysregulation transitions the repair trajectory away from functional regeneration and irreversibly toward fibrotic scarring. It is critical to emphasize that fetal scarless healing does not merely represent an amplified adult response but instead reflects a qualitatively distinct immunomechanical state.

Reduced inflammatory amplitude, differential TGF-β isoform balance, and diminished mechanotransductive signaling define a developmentally programmed regenerative phenotype. Accordingly, regenerative strategies should aim to restore this qualitative microenvironmental configuration rather than simply amplify the proliferative output in aged tissues.

### 7.2 The regenerative landscape: a multidimensional framework of exhaustion

**FIGURE 4:**
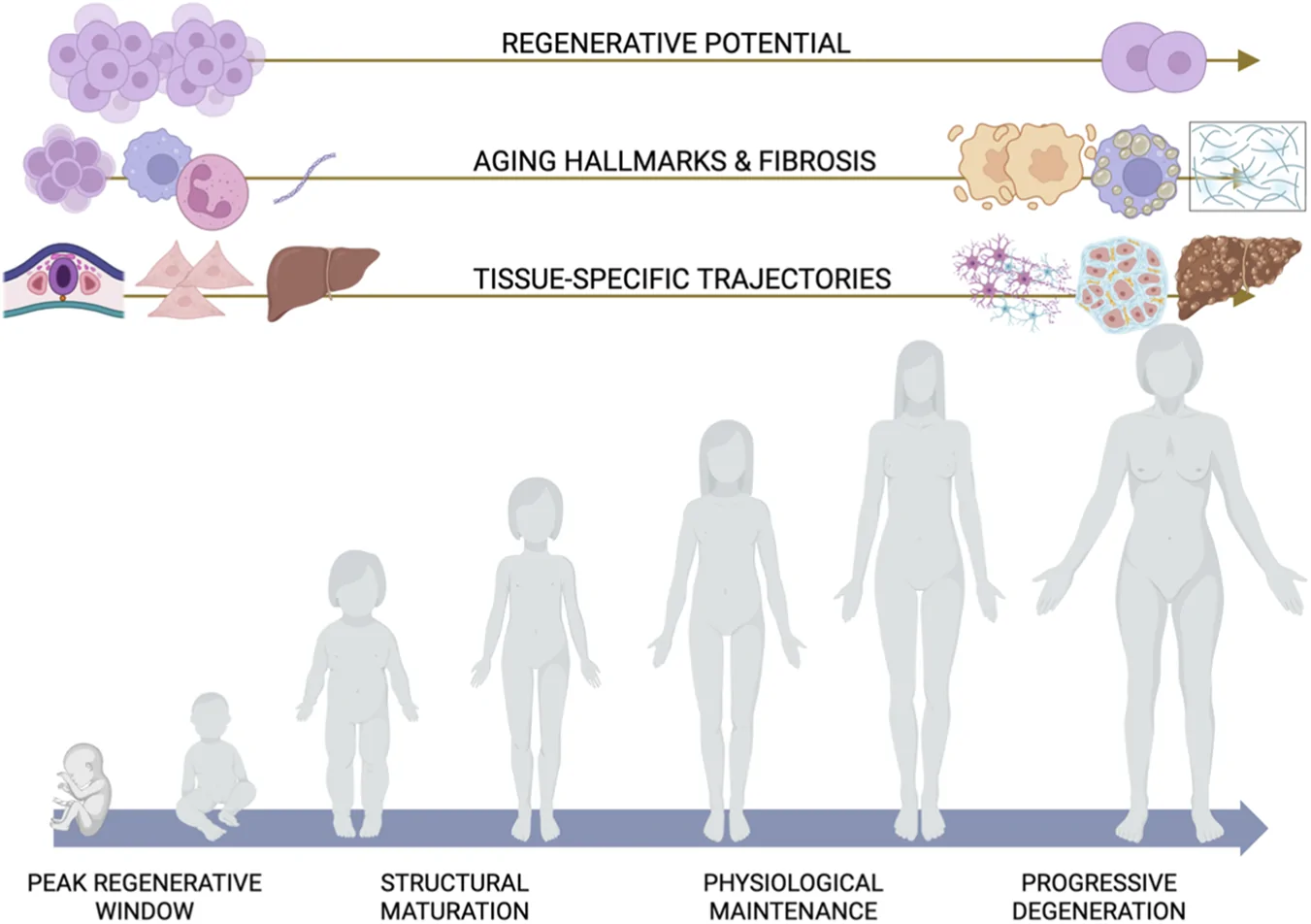
Ontogenetic landscape of human tissue repair. This schematic illustrates the systemic transition in regenerative capacity across the human lifespan. Top: embryonic and neonatal stages are characterized by high plasticity and robust stem cell pools, facilitating scarless repair or complete functional restoration, such as neurogenesis and cardiomyocyte proliferation. Bottom: the transition toward adulthood and senescence is marked by the progressive accumulation of aging hallmarks, including stem cell exhaustion, inflammaging, and epigenetic drift. Middle: these changes lead to divergent organ-specific trajectories, in which tissues lose regenerative competence and increasingly default to fibrotic scarring and functional decline, including glial scarring, cardiac fibrosis, and liver cirrhosis.

Tissue repair and regenerative capacity vary markedly across the human lifespan, reflecting a complex interplay among genetic, epigenetic, systemic, and environmental networks (López-Otín et al., 2023; Feil and Fraga, 2012). Understanding this ontogenetic continuum—from the flawless plasticity of the embryo to the multidimensional exhaustion of older age—provides the critical biological framework needed to develop targeted regenerative therapies (Figure 4).

During embryogenesis, DNA repair systems operate with maximal fidelity alongside extensive epigenetic reprogramming, ensuring genomic stability and scarless tissue formation (Wilkinson et al., 2023). Childhood and adolescence represent a window of heightened plasticity, during which robust stem cell populations and specialized neurogenic and cardiogenic windows facilitate rapid recovery from injury (Kuipers et al., 2015).

However, as organisms transition through adulthood into senescence, this highly coordinated matrix deteriorates. The accumulation of unresolvable DNA damage, telomere attrition, and mitochondrial dysfunction establishes a foundation of genomic instability. Simultaneously, epigenetic drift and inflammaging dismantle stem cell niches, creating a hostile microenvironment that favors fibrotic scarring over functional regeneration (Franceschi et al., 2018; Levine et al., 2018). This systemic and localized decline culminates in multidimensional exhaustion during older age, a progressive functional loss that highlights the unique molecular and clinical barriers present at each stage of the human lifespan (Table 3).

**TABLE 3:** Differential regenerative potential and molecular regulators across lifespan.

Organ system	Embryonic/neonatal potential	Adult regenerative capacity	Key molecular regulators	Primary clinical barriers
Central nervous system	High axonal plasticity; absence of inhibitory scarring	Extremely limited; restricted to specific niches (SVZ/SGZ)	DMTF1, NogoA, MAG, and OMgp	Glial scar; myelin-associated inhibitors; stem cell exhaustion
Myocardium	Robust cardiomyocyte proliferation; functional recovery	Minimal; predominantly replaced by fibrotic scar tissue	Hippo/YAP, Neuregulin-1, and ErbB4	Persistent fibrotic transition; cardiomyocyte terminal differentiation
Hepatic tissue	Rapid, high-fidelity mass restoration	High (compensatory) but finite under chronic stress	Wnt/β-catenin, IL-6/STAT3, and Hippo/YAP.	Inflammaging; HPC exhaustion
Cutaneous tissue	Scarless healing; perfect dermal architecture	Reparative (not regenerative); results in fibrotic scarring	IL-10, TGF-β isoforms, and collagen III:I ratio	Dysregulated macrophage polarization; myofibroblast activity

HPC: hepatic progenitor cell; SGZ: subgranular zone; SVZ: subventricular zone.

## 8 Geroscience in clinical practice: a concise evidence-based framework for biological aging assessment and healthspan optimization

The hallmarks of aging provide a mechanistic framework for understanding age-related morbidity; however, clinical geroscience must also address two operational imperatives: the reproducible quantification of biological aging and the translation of biomarker variation into clinically meaningful outcomes (López-Otín et al., 2023). Although important mechanistic insights are derived from model organisms, the practical goal of geroscience is to determine how aging biology manifests in human physiology, chronic disease trajectories, functional decline, and resilience and how these processes can be measured reliably.

This distinction is clinically important because not every age-associated biological change should be interpreted as primarily pathological. Some changes may initially represent adaptive or compensatory responses that preserve physiological homeostasis under cumulative oxidative, inflammatory, metabolic, or mechanical stress. Over time, however, these same responses may become insufficient, persistent, or maladaptive. A clinically useful geroscience framework, therefore, requires a disciplined distinction between adaptive remodeling and pathological deterioration, while retaining physiological homeostasis—not biomarker normalization alone—as the principal objective.

This review integrates current evidence across measurement strategies, pharmacological interventions, lifestyle medicine, and emerging modalities, maintaining a deliberate separation between established findings and investigational hypotheses. Throughout, the emphasis is not merely on identifying biological age signals but on determining whether those signals translate into function, resilience, and actionable clinical outcomes.

### 8.1 Measuring biological aging

#### 8.1.1 Clocks of biological aging

Biological aging assessment now relies on a hybrid framework that combines molecular clocks with functional phenotyping. DNA methylation-based tools have evolved from chronological age predictors toward morbidity-linked instruments; DunedinPACE offers improved precision as a longitudinal pace-of-aging biomarker, although methylation clocks remain intermediate measures rather than validated clinical surrogates for reducing clinical events (Belsky et al., 2022; Hannum et al., 2013; Horvath, 2013).

Proteomic aging clocks, in turn, leverage circulating protein signatures to predict mortality across diverse populations. IgG glycosylation patterns also capture immune remodeling relevant to aging-related disease states (Argentieri et al., 2024; Wu et al., 2024).

These tools are promising, but they should not be interpreted in isolation. Their clinical value depends on whether they reflect meaningful biological vulnerability rather than statistical association with age alone. For this reason, all molecular biomarkers should be anchored by functional phenotyping—frailty indices, grip strength, gait speed, and cardiorespiratory fitness proxies—because function is patient-centered, strongly prognostic, and directly actionable (López-Otín et al., 2023). In clinical geroscience, a marker gains importance not because it changes with age but because it clarifies risk, tracks physiological reserve, or helps guide intervention.

#### 8.1.2 The gut microbiome as a convergence node

Age-associated dysbiosis, reduced microbial diversity, increased intestinal permeability, and downstream immune activation provide a mechanistic pathway to chronic low-grade inflammation and amplified age-related vulnerability (Gyriki et al., 2025). In this respect, the gut microbiome may be viewed less as an isolated domain and more as a convergence node linking nutrient sensing, immune tone, barrier integrity, and systemic metabolic signaling. Microbiome-directed strategies, therefore, represent a rational investigational complement to established interventions targeting the hallmarks of aging, although large-scale outcome evidence remains limited.

### 8.2 Nutrient-sensing pathways and metabolic interventions

Chronic mTORC1 overactivation suppresses autophagy and reinforces anabolic signaling at the expense of cellular maintenance. Rapamycin robustly extends lifespan in mammalian models, and low-dose mTOR-pathway inhibition in humans has improved influenza vaccine responses and reduced infection rates in older adults (Mannick et al., 2014; 2018; Saxton and Sabatini, 2017; Harrison et al., 2009). These findings support mTOR as one of the most appealing translational targets in geroscience, although the boundary between mechanistic plausibility and durable clinical benefit remains important and under active investigation.

Metformin remains appealing because of its effects on AMPK activation, mitochondrial complex I, and downstream metabolic regulation. Meta-analytic data associate it with reduced all-cause mortality, but generalizability to healthy non-diabetic individuals still awaits adequately powered randomized trials (Anisimov, 2010; Barzilai et al., 2016; Campbell et al., 2017). Senolytic agents target SASP-driven tissue dysfunction by selectively eliminating senescent cells; first-in-human studies indicate biological activity, but clinical use outside investigational settings remains premature (Kirkland and Tchkonia, 2020; Justice et al., 2019).

Conceptual caution is warranted in this area. Senescent cells, when excessive, are clearly harmful; however, the relationship among senescent-cell persistence, apoptosis resistance, inflammation, and tissue dependence requires deeper study (Burton and Krizhanovsky, 2014). Their elimination may be beneficial, but the consequences are likely to be tissue-specific and context-dependent. The clinical question is, therefore, not whether senescent cells are always harmful or always protective but when their persistence becomes more damaging than their removal.

Taurine and alpha-ketoglutarate show mechanistic coherence and promising preclinical data, but definitive human outcome evidence remains absent; therefore, they warrant cautious interest rather than premature clinical adoption (Asadi Shahmirzadi et al., 2020; Demidenko et al., 2021; Singh et al., 2022). GLP-1 receptor agonists merit inclusion in a longevity-oriented framework because of their established cardiovascular benefit in relevant populations and their favorable effects on adiposity, glycemic burden, blood pressure, and inflammatory tone (Lincoff et al., 2023; Liuzzo and Patrono, 2024). In contrast, aspirin illustrates the opposite problem: plausible anti-inflammatory logic is outweighed by clinically meaningful bleeding risk, and current evidence does not support its use as a general longevity agent in healthy older adults (Gaziano et al., 2018; McNeil et al., 2018; US Preventive Services Task Force, 2022).

### 8.3 Systemic geroscience: modulating the hallmarks of aging

#### 8.3.1 Lifestyle foundations: the primary geroprotectors

Exercise remains the most consistently supported multisystem geroprotective intervention. It modulates nutrient sensing, mitochondrial function, endothelial health, insulin sensitivity, and inflammatory tone while preserving muscle mass and physical reserve. Resistance training is particularly important for buffering sarcopenia, frailty trajectories, and downstream disability, with meaningful epidemiological mortality benefit at moderate volumes (Arem et al., 2015).

Sleep regularity has emerged as a stronger predictor of mortality risk than sleep duration, reinforcing the clinical importance of stable sleep–wake timing over total hours alone (Cappuccio et al., 2010; Windred et al., 2024; Yin et al., 2017). Loneliness, social isolation, and living alone are independently associated with increased mortality, justifying the integration of social context into healthspan frameworks as both a neuroendocrine stress determinant and an adherence mediator for physical activity and preventive care (Holt-Lunstad et al., 2015). Taken together, these variables underscore that biological aging is not only molecularly embodied but also behaviorally and socially shaped.

#### 8.3.2 Autonomic aging and heart-rate variability

Age-associated reduction in vagal tone, together with relative sympathetic predominance, links chronic stress, sleep disruption, systemic inflammation, and impaired recovery. Heart-rate variability is a pragmatic, non-invasive signal of autonomic strain when measured under standardized conditions, but it should not be treated as an independent clinical endpoint. Its value lies in contextual interpretation, particularly when integrated with sleep, exercise tolerance, psychosocial burden, and inflammatory status.

#### 8.3.3 Adjunct modalities

Sauna bathing has been prospectively associated with reduced cardiovascular and all-cause mortality, although causality remains debated; it is best positioned as a recovery adjunct rather than a core geroscience intervention (Laukkanen et al., 2015). Photobiomodulation in red and near-infrared wavelengths is mechanistically plausible for modulating mitochondrial function and supporting muscle recovery, but heterogeneous clinical evidence precludes its designation as a foundational longevity intervention pending adequately powered trials in aging populations (Ferraresi et al., 2016; Kumar et al., 2024; Nascimento et al., 2024). Structured music interventions show consistent signals for improving heart-rate variability and sleep quality in stressed or sleep-disordered populations; however, specific claims attributing anti-aging effects to solfeggio frequencies remain unsupported by clinical evidence (Zhang et al., 2026; Zhao et al., 2024).

#### 8.3.4 Redox control, RNA vulnerability, and mitochondrial DNA

Redox biology deserves a central place in any geroscience framework. The field has moved beyond a simplistic model in which reactive oxygen species are treated only as toxic by-products of metabolism. Reactive oxygen species also act as pleiotropic signaling agents that regulate adaptation, inflammation, mitochondrial function, and cell fate (Sies and Jones, 2020). The challenge is not to deny oxidative injury but to identify the transition from adaptive redox signaling to pathological oxidative dysregulation.

Although DNA damage remains a major feature of aging biology, RNA is also highly susceptible to oxidative injury. RNA is predominantly single-stranded, broadly distributed in the cytoplasm, and lacks both histone protection and the stabilizing architecture of double-stranded chromatin. Oxidative RNA damage may impair translation, ribosomal fidelity, and stress-responsive protein synthesis, potentially affecting cellular function before irreversible genomic damage becomes evident (Li et al., 2006).

Mitochondrial DNA also deserves a separate emphasis. Although mtDNA is organized in nucleoids and associated with proteins such as TFAM, it is not protected by canonical histones and resides near the electron transport chain, where reactive oxygen species are continuously generated. This structural and metabolic context contributes to its distinctive vulnerability and helps explain why mitochondrial dysfunction occupies such a central position in aging biology (Alexeyev et al., 2013).

#### 8.3.5 Mitochondrial quality control and epigenetic support

Mitochondrial decay severely impairs high-energy tissues. Urolithin A (UA), a microbiome-derived metabolite from ellagitannins, acts as a first-in-class inducer of mitophagy, facilitating the selective recycling of dysfunctional mitochondria (Ryu et al., 2016). Clinical trials confirm that UA is safe and improves muscle performance and mitochondrial biomarkers in middle-aged adults, offering a direct intervention against functional decline (Andreux et al., 2019; Singh et al., 2022). This mitochondrial resilience is closely linked to epigenetic regulation via longevity-associated genes. Sirtuin 1 (*SIRT1*), a nicotinamide adenine dinucleotide (NAD+)-dependent deacetylase, links nutrient availability to DNA repair and mitochondrial biogenesis (Sugishita et al., 2024). In particular tissues, SIRT1 restrains catabolic pathways; its loss accelerates osteoarthritis, positioning sirtuin-linked therapies as rational adjuncts for chondrocyte proteostasis (Matsushita et al., 2013).

The genetic architecture of longevity also highlights the stress-resistant *FOXO3* gene and the vasculoprotective *KLOTHO* gene, whose encoded circulating protein may be positively influenced by aerobic exercise (Willcox et al., 2008; Martins et al., 2016; Kuro-o et al., 1997; Saghiv et al., 2017). Additionally, dietary polyphenols exert significant systemic effects; evidence from the Ohsaki Study indicates that consuming five or more cups of green tea per day is inversely associated with cardiovascular and all-cause mortality, likely mediated by catechins such as epigallocatechin gallate (EGCG), which improve endothelial function (Kuriyama et al., 2006; Potenza et al., 2007).

#### 8.3.6 Senotherapeutics and emerging longevity metabolites

A critical barrier to tissue regeneration is the accumulation of senescent cells secreting a pro-inflammatory SASP. Senolytic agents aim to selectively induce apoptosis in these cells (Kirkland and Tchkonia, 2020). First-in-human pilot studies utilizing intermittent dasatinib plus quercetin (D + Q) have shown feasibility and signals of improved physical function in idiopathic pulmonary fibrosis, paving the way for clinical senolysis (Justice et al., 2019).

Furthermore, emerging metabolites show significant promise. Taurine supplementation has been shown to extend the lifespan and improve aging phenotypes in animal models (Singh et al., 2025), while alpha-ketoglutarate (AKG), a Krebs cycle intermediate, has demonstrated morbidity compression. Notably, retrospective analyses of humans consuming AKG-containing supplements reported reductions in DNA methylation (DNAm) biological age clocks, though prospective validation remains necessary (Asadi Shahmirzadi et al., 2020; Demidenko et al., 2021).

Although these approaches remain in the preclinical sphere, studies in mouse models have shown that selectively eliminating senescent cells through genetic, pharmacological, or gene-therapy strategies—including CAR-T cells targeting senescent cells—that induce apoptosis (senoptosis) can extend the lifespan and improve multiple age-related conditions (Chang et al., 2016; de Keizer et al., 2010; Zhu et al., 2016).

#### 8.3.7 Climate change, biological resilience, and healthy aging

Climate gerontology extends clinical geroscience from the measurement of biological aging to the environmental conditions that increasingly determine whether repair capacity can be preserved in later life. Climate change affects older adults not only through acute mortality during disasters but also by constraining intrinsic capacity, functional ability, and the social environments that support recovery (Prina et al., 2024; Zhang et al., 2025). Heat exposure, ambient air pollution, wildfire smoke, drought, displacement, sleep disruption, dehydration risk, and reduced access to cooling or health services should, therefore, be considered clinically relevant stressors within a modern geroscience framework (Figueiredo et al., 2024; Prina et al., 2024; Montoro-Ramírez et al., 2025; Zhang and Chen, 2024).

These conditions converge on several biological systems emphasized throughout this review. Extreme heat increases cardiovascular and renal workload, promotes dehydration, worsens orthostatic instability, and may amplify frailty trajectories in individuals with limited physiological reserve (Figueiredo et al., 2024; Prina et al., 2024; Montoro-Ramírez et al., 2025). Air pollution and wildfire smoke intensify oxidative stress, endothelial dysfunction, autonomic strain, inflammatory signaling, and respiratory vulnerability; emerging evidence also links air pollution exposure with increased frailty risk in middle-aged and older adults (Figueiredo et al., 2024; Jafari et al., 2025; Montoro-Ramírez et al., 2025). In parallel, climate-related sleep fragmentation, circadian disruption, and nighttime heat exposure may impair immune calibration, metabolic regulation, cognitive performance, and tissue repair, thereby reducing the margin between adaptation and decompensation.

The biological relevance of climate exposure is increasingly supported by molecular aging data. Ambient outdoor heat has been associated with accelerated epigenetic aging among older adults, indicating that environmental stress may become measurable not only as morbidity or mortality, but also as a change in biological age signals (Choi and Ailshire, 2025). This does not yet establish heat exposure as a validated clinical surrogate for age-related disease, but it strengthens the rationale for integrating exposomic assessment with methylation clocks, frailty indices, vascular metrics, sleep regularity, hydration status, cognitive screening, and functional phenotyping.

A climate-aware geroscience model should, therefore, treat resilience as both a cellular property and a population-level capacity. At the cellular level, resilience reflects the ability to preserve proteostasis, mitochondrial function, vascular responsiveness, immune calibration, and repair after thermal, oxidative, inflammatory, or circadian stress. At the clinical level, it appears as preserved gait speed, muscle strength, cognition, hydration stability, medication safety, sleep–wake regularity, and recovery after illness or environmental exposure. At the societal level, it depends on housing quality, access to cooling, clean air, social connection, disaster preparedness, mobility support, and age-friendly public-health systems (Figueiredo et al., 2024; Prina et al., 2024; Zhang et al., 2025). Thus, climate gerontology provides an essential bridge between molecular geroscience and real-world vulnerability: the same biological pathways that define aging are increasingly shaped by the environments in which older adults live, recover, and attempt to maintain independence.

### 8.4 Targeted regenerative medicine and organ-specific bioengineering

While systemic geroscience optimizes the global physiological environment, advanced regenerative medicine addresses the localized failure of specific organs. As established previously, the liver replaces, the brain rewires, and the adult heart scars.

Therefore, modern clinical strategies must respect these distinct biological landscapes. In the cardiovascular system, bypassing the post-mitotic arrest of adult cardiomyocytes requires targeted genetic and molecular reactivation. Experimental interventions, such as the reactivation of the Cyclin A2 (*CCNA2*) gene, have successfully induced mitotic division in adult human heart cells (Bouhamida et al., 2025). Similarly, modifying calcium handling by inhibiting the L-Type Calcium Channel (LTCC) alters calcineurin activity to stimulate cardiomyocyte replication, while novel mRNA-based therapies—such as phosphoserine aminotransferase 1 (*PSAT1*) modified mRNA (modRNA)— demonstrate robust increases in proliferation and reduced scarring in animal models (Feng et al., 2026; Magadum et al., 2025). Concurrently, cell-free therapies utilizing stem cell-secreted extracellular vesicles (stem-EVs) are emerging as potent alternatives to direct MSC or iPSC grafts. These vesicles modulate immune responses and promote angiogenesis without the risks of cell rejection or arrhythmias sometimes associated with whole-cell engraftment (Karim Rony and Tompkins, 2025).

In the central nervous system, where structural replacement is anatomically restricted, the therapeutic focus transitions to enhancing neuroplasticity and functional rewiring. Gene editing tools, notably clustered regularly interspaced short palindromic repeats (CRISPR)-associated protein 13 (Cas13), are being utilized to correct pathogenic mutations and suppress toxic gene expression (such as by RNA-targeting Cas systems) to restore neuronal function (Yashooa et al., 2026). Furthermore, identifying transcription factors such as cyclin D-binding myb-like transcription factor 1 (DMTF1) offers novel pathways to regulate and rejuvenate neural stem cell activity in the aging brain; this is complemented by the role of Stathmin-2, a critical microtubule-associated protein. Recent evidence highlights that the loss of STMN2, often driven by age-related proteinopathies and TAR DNA-binding protein 43 (TDP-43) dysfunction, severely impairs axonal outgrowth and regenerative fidelity, marking it as a key target for restoring neural integrity (Liang et al., 2026; Glass, 2020). To actively overcome the physical and molecular barriers formed by glial scars and myelin-associated inhibitory proteins—including NogoA, myelin-associated glycoprotein (MAG), and oligodendrocyte myelin glycoprotein (OMgp)—modern neuro-regenerative approaches integrate biomaterial scaffolds designed for the localized infusion of neurotrophic factors such as BDNF, NGF, IGF-I, and leukemia inhibitory factor (LIF). These molecular interventions are increasingly synergized with artificial intelligence (AI)-assisted neurorehabilitation and precision diagnostics to map and maximize synaptic connectivity during recovery (Tataranu and Rizea, 2025).

Conversely, given the liver’s unparalleled intrinsic regenerative capacity, clinical efforts in hepatology diverge from replacing tissue to prevent the exhaustion caused by chronic inflammation. Antifibrotic therapies and the precise modulation of developmental pathways—specifically Wnt/β-catenin, IL-6/STAT3, and Hippo/YAP—aim to preserve hepatic progenitor cell (HPC) activity and block the fibrotic transition to irreversible cirrhosis (Ye et al., 2025). Ultimately, regeneration is not a one-size-fits-all process. The future of regenerative medicine lies in combining the systemic rejuvenation provided by geroscience with targeted, organ-specific molecular and cellular interventions, harnessing the residual strengths of each tissue while overcoming its acquired ontogenetic limitations.

Nevertheless, translational rejuvenation strategies must be implemented with precision. Reactivating proliferative or epigenetic programs carries an inherent oncogenic risk if the tumor suppressive safeguards are destabilized. The next-generation of regenerative interventions must, therefore, balance repair activation with genomic stability, ensuring the restoration of function without compromising long-term cellular integrity.

### 8.5 Frontier: partial epigenetic reprogramming

OSK-based (Oct4, Sox2, and Klf4) partial reprogramming strategies show compelling preclinical evidence of functional restoration, but unresolved challenges in delivery, durability, and oncogenic risk place these approaches firmly within the investigational frontier rather than current clinical practice. Their importance may ultimately lie less in immediate application than in conceptual impact. If age-related dysfunction is even partially reversible at the level of cellular state rather than only suppressible downstream, reprogramming could mark a major change in how chronic age-related disease is approached (López-Otín et al., 2023; Lu et al., 2020).

### 8.6 Risks, ethics, and responsible implementation

Polypharmacy, supplement quality variability, biomarker chasing without validated surrogate linkage, and inequitable access to diagnostics represent concrete clinical risks in longevity medicine (Kirkland and Tchkonia, 2020; López-Otín et al., 2023). A mature informed-consent model requires explicit differentiation among established, promising, and investigational interventions, consistently aligning patient expectations with the actual strength of available evidence (see Table 4 for a detailed evidence mapping of common interventions in humans). This distinction is particularly important in a field where mechanistic enthusiasm can easily outpace clinical certainty.

**TABLE 4:** Evidence mapping for common interventions in humans.

Intervention	Evidence level	Key outcomes	Main limitations
Structured physical activity	Observational large, pooled cohorts; RCTs for function	Lower mortality risk association; improved function/frailty markers	Confounding in mortality cohorts; heterogeneity in protocols
Sleep duration/regularity targets	Observational meta-analyses; cohort actigraphy	Mortality/cardiometabolic risk associations; regularity predicts mortality	Reverse causality; residual confounding; behavior measurement error
Social connectedness	Observational meta-analysis	Mortality risk association	Confounding; measurement heterogeneity
mTOR/TORC1 pathway modulation (low dose)	RCTs; immune endpoints	Improved influenza vaccine response; fewer infections reported	Not powered for mortality; long-term safety/durability uncertain; off-label risk
Metformin	Meta-analysis primarily observational comparisons; established cardiometabolic outcomes	Lower mortality/disease associations in treated populations	Confounding; unclear generalizability to healthy non-diabetics; TAME not outcome complete
Urolithin A	Human safety trials; RCTs functional/biomarker endpoints	Safety, improved muscle performance measures, and mitochondrial-related biomarkers	Limited duration; endpoints intermediate; population/task specificity
Senolytics (e.g., dasatinib + quercetin)	First-in-human pilot open-label; reviews	Feasibility: preliminary functional signals in IPF	No definitive efficacy/safety; target specificity unresolved; investigational
GLP-1 receptor agonist	RCT hard outcomes	Reduced MACE in obesity without diabetes and established CVD.	Population-specific; no general longevity proof; tolerability/adherence issues
Aspirin (primary prevention)	RCTs; guideline synthesis	No disability-free survival benefits the healthy elderly; increased bleeding	Net benefit varies by age/risk; harms limit broad use
Taurine	Preclinical; limited human translation	No validated aging-outcome benefit	Dose/endpoint uncertainty; trial gap
Alpha-ketoglutarate	Preclinical; non-randomized biomarker observations	No validated clinical endpoint benefit	Biomarker-only human data; commercial formulation confounding; needs RCTs

Aging is a partially modifiable biological process governed by conserved, overlapping pathways that respond to nutrition, physical activity, sleep–wake regularity, psychosocial context, redox balance, immune regulation, targeted pharmacological intervention, and increasingly relevant environmental exposures that shape biological resilience (López-Otín et al., 2023; Prina et al., 2024; Zhang and Cheng, 2024; Zhang et al., 2025; Montoro-Ramírez, et al., 2025; Figueiredo et al., 2024; Choi and Ailshire, 2025; Jafari et al., 2025). Yet not every age-related change is inherently pathological. Some changes reflect adaptive attempts to preserve homeostasis under progressively constrained conditions.

Rigorous clinical practice in geroscience, therefore, demands the integration of mechanistic evidence and clinical outcomes, explicit acknowledgment of uncertainty, and an unwavering commitment to patient-centered functional outcomes over biomarker optimization alone. Under that discipline, geroscience can move beyond promise toward a clinically responsible framework for healthspan optimization that includes both biological repair and protection of the environment in which resilience is either preserved or depleted.

## 9 Discussion

Advances in next-generation sequencing, single-cell multi-omics, and spatial transcriptomics are providing high-resolution insights into the molecular dynamics of tissue repair across life stages. In the embryonic stage, these technologies reveal how tightly coordinated epigenetic regulation supports developmental fidelity, offering a molecular reference for biomimetic tissue repair and regenerative medicine. However, translation requires caution: developmental programs cannot simply be reactivated wholesale in aged tissues without accounting for tumor suppression, immune surveillance, tissue context, and long-term functional consequences (Wilkinson et al., 2023).

In later life stages, the research frontier is transitioning toward strategies that may partially restore youthful repair competence in aged tissues. *In vivo* epigenetic reprogramming—through transient and controlled expression of pluripotency-associated factors—has shown preclinical potential to reverse selected epigenetic age signals, improve cellular function, and enhance tissue resilience while preserving cell identity (Ocampo et al., 2016; Lu et al., 2020; Yousefzadeh et al., 2021). These findings are conceptually important, but they remain investigational and should not be interpreted as evidence of established clinical rejuvenation.

Concurrently, the therapeutic mRNA era offers a plausible route for targeted, time-limited intracellular delivery of transcription factors or deficient proteins to support local homeostasis. The central translational challenge is to define a precise therapeutic window: interventions must be strong enough to reactivate dormant repair pathways or delay functional decline, yet they must be sufficiently controlled to avoid unregulated proliferation, loss of cell identity, immune toxicity, or oncogenesis.

Ultimately, tissue repair is not a static biological parameter; it is a dynamic, ontogenetically determined continuum shaped by genomic integrity, cellular plasticity, systemic regulation, and environmental context. Early developmental stages—from the embryo to childhood—are characterized by high-fidelity, plastic, and often scarless repair mechanisms. Conversely, progression into adulthood and senescence is marked by a progressive decline in regenerative competence. This deterioration is not a single point of failure but a systems-level loss of resilience driven by accumulating DNA damage, epigenetic drift, stem cell exhaustion, mitochondrial dysfunction, and chronic inflammaging (Table 2).

Understanding the molecular, cellular, systemic, and environmental determinants that drive the loss of plasticity across the lifespan provides a roadmap for translational medicine. Modern therapeutic strategies—ranging from *in vivo* gene editing and epigenetic modulation to senolytic clearance, extracellular vesicle therapies, and targeted mRNA delivery—aim to restore permissive repair environments. Their clinical value, however, will depend on whether they improve function, resilience, safety, and meaningful healthspan outcomes rather than biomarkers alone.

Collectively, these approaches represent a paradigm shift from passively managing chronic degeneration toward actively preserving or restoring endogenous repair capacity. This integration of systemic geroscience, targeted pharmacological intervention, lifestyle and environmental resilience strategies, and organ-specific bioengineering can be conceptualized as a “longevity staircase,” in which each therapeutic tier builds on the previous one to support functional healthspan while respecting the limits of current evidence.

**FIGURE 5:**
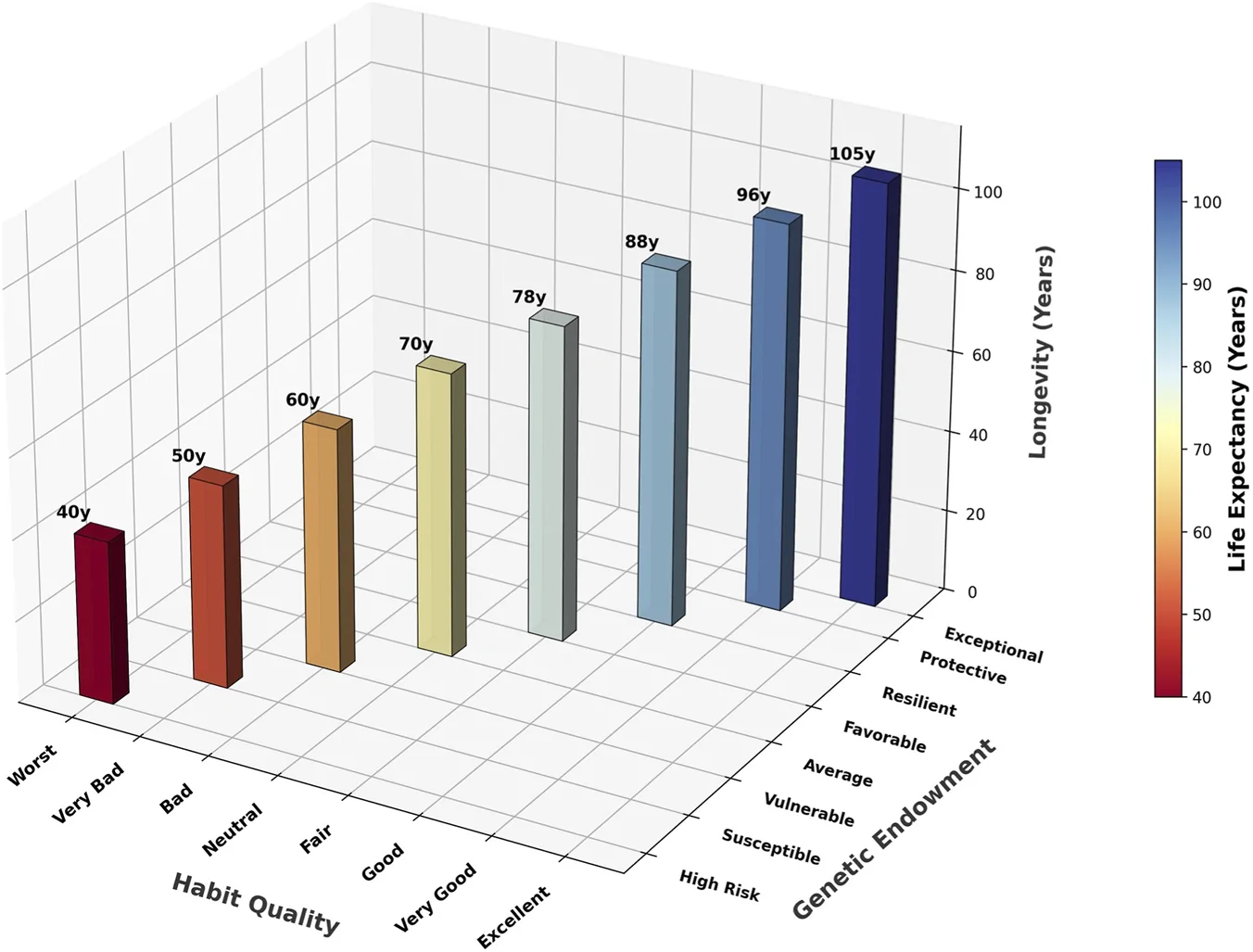
Longevity staircase: synergistic impact of intrinsic repair potential, lifestyle, environmental modifiers, and evidence-aligned interventions on human healthspan and biological resilience.

The three-dimensional conceptual model illustrated in Figure 5 depicts healthspan trajectories shaped by the interaction between intrinsic cellular repair capacity and extrinsic modifiers, including lifestyle, environmental exposure, and access to supportive care. The most favorable trajectory occurs when preserved genetic and epigenetic repair networks are reinforced by high-quality habits, risk reduction, and evidence-aligned geroprotective strategies. Conversely, poor lifestyle conditions, adverse environmental exposures, and the natural ontogenetic decline of repair mechanisms may accelerate systemic deterioration and reduce resilience.

Mastering the ontogenetic rules of tissue repair may help preserve organ function, reduce the burden of age-related degenerative disease, and extend functional healthspan. Achieving this goal will require the same discipline emphasized in clinical geroscience: prioritizing physiological resilience, validated functional outcomes, safety, equity, and transparent distinction between established interventions and investigational frontiers.
